# Inhalable microparticles as drug delivery systems to the lungs in a dry powder formulations

**DOI:** 10.1093/rb/rbac099

**Published:** 2022-12-08

**Authors:** Karolina Knap, Konrad Kwiecień, Katarzyna Reczyńska-Kolman, Elżbieta Pamuła

**Affiliations:** Department of Biomaterials and Composites, Faculty of Materials Science and Ceramics, AGH University of Science and Technology, 30-059 Krakow, Poland; Department of Biomaterials and Composites, Faculty of Materials Science and Ceramics, AGH University of Science and Technology, 30-059 Krakow, Poland; Department of Biomaterials and Composites, Faculty of Materials Science and Ceramics, AGH University of Science and Technology, 30-059 Krakow, Poland; Department of Biomaterials and Composites, Faculty of Materials Science and Ceramics, AGH University of Science and Technology, 30-059 Krakow, Poland

**Keywords:** pulmonary therapies, drug delivery systems to lungs, microparticles, inhalers, dry powder inhalers

## Abstract

Inhalation-administrated drugs remain an interesting possibility of addressing pulmonary diseases. Direct drug delivery to the lungs allows one to obtain high concentration in the site of action with limited systemic distribution, leading to a more effective therapy with reduced required doses and side effects. On the other hand, there are several difficulties in obtaining a formulation that would meet all the criteria related to physicochemical, aerodynamic and biological properties, which is the reason why only very few of the investigated systems can reach the clinical trial phase and proceed to everyday use as a result. Therefore, we focused on powders consisting of polysaccharides, lipids, proteins or natural and synthetic polymers in the form of microparticles that are delivered by inhalation to the lungs as drug carriers. We summarized the most common trends in research today to provide the best dry powders in the right fraction for inhalation that would be able to release the drug before being removed by natural mechanisms. This review article addresses the most common manufacturing methods with novel modifications, pros and cons of different materials, drug loading capacities with release profiles, and biological properties such as cytocompatibility, bactericidal or anticancer properties.

## Graphical Abstract

**Figure:**
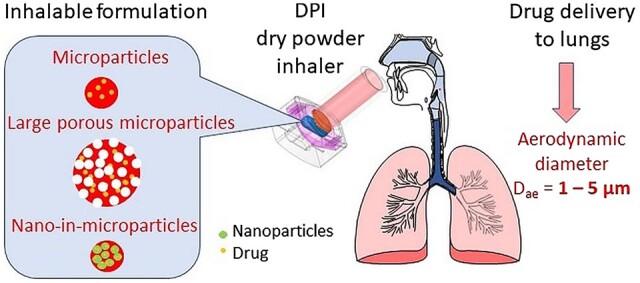

## Introduction

Decades of research in the field of medicine and pharmacy resulted in the development of numerous drugs and active pharmaceutical ingredients (API). Long-lasting studies, hundreds if not thousands of case reports and meta-analyses, experiences and treatment outcomes of millions of patients worldwide, provided invaluable insight into drug mode of action, efficacy, safety and toxicology profiles. Having understood what drug limitations in the use are or what the major source of risk of the drug’s failure is, it is possible to develop a drug delivery system (DDS) using an already existing and approved for use drugs in a novel formulation.

According to the National Institutes of Health (NIH), DDSs can generally be described as engineered devices designed for the targeted delivery or controlled release of active components [1]. In most cases, the drug is encapsulated within a biocompatible shell, providing protection against premature degradation due to the presence of both physicochemical and biological factors [2]. Drug carriers may consist of different materials, such as natural or synthetic polymers, lipids, metal oxides or metals [3]. The main advantages of DDSs include improved bioavailability, prolonged drug circulation, control over drug release kinetics and a reduced risk of negative side effects. Modern technologies allow for various modifications of DDSs, functionalization or sensitization to different stimuli to further increase the efficacy of treatment [2, 4]. Another important issue is related to the cost effectiveness of the development of DDSs based on already existing drugs compared to the development of a new drug (new molecular entity, NME). Considering the 10% success rate of NME in clinical trials and knowing that NME costs are increasing on average 13.4% per year, pharmaceutical companies are more likely to turn to advanced DDS [5]. The carriers in DDSs may deliver drugs in a different way: transdermal (e.g. membrane), implantable (e.g. stents, implants), parenteral (e.g. hypodermic needles) or pulmonary (e.g. liposomes, solid lipid and polymer particles) [6–8].

Pulmonary DDSs remain the most popular [5]. The large surface area of the lungs (around 100 m^2^), excellent vascularization, and relatively mild environment (in terms of the presence of immune cells, enzymes or metabolic reactions) offer a unique opportunity to deliver drugs locally and systematically [9, 10]. In the case of local drug administration, high doses are delivered directly to the action site; thus, the same therapeutic effect can be obtained with the use of a significantly lower drug dose in comparison to, e.g. oral or intravenous administration. In terms of systemic treatment, drugs administered by inhalation are rapidly absorbed into the bloodstream, which not only decreases the time between administration and the onset of action, but also reduces the risk of drug inactivation (e.g. during liver first-pass metabolism) [9].

Inhalation therapy for lung diseases, especially asthma, was first recognized in India around 2000 DC by Ayurvedic medicine and was based on smoked herbs (namely datura roots) that were later found to contain bronchodilating alkaloids. Later examples of inhalable therapies were found in ancient Egypt and Greece, followed by, e.g. medieval Spain. The breakthroughs of the 18th and 19th centuries in medicine resulted in the development of modern ceramic inhalers or nebulizers utilizing medicated vapors or steam. The first reports on inhalable drug delivery date back to 1910, when a bronchodilator, epinephrine, was used in the form of an aerosol. Remarkable improvements in pulmonary delivery were made in the mid-20th century when Riker Labs (currently 3M Pharmaceuticals) introduced the first pressurized meter dose inhaler (pMDI)—a device that allowed for more precise control over the inhaled dose and was more convenient for patients [11, 12]. PMDIs were initially designed for patients with asthma and chronic obstructive pulmonary disease (COPD) and were used to deliver isoproterenol or epinephrine [9]. Dry powder inhalers (DPIs) were first developed in the 1970s of the 20th century [11].

Nowadays, inhalable formulations of various bronchodilators or corticosteroids are available for patients suffering from respiratory diseases such as asthma and COPD [13, 14]. Systems of pulmonary administration of anti-infective drugs (i.e. tobramycin (Tobr), amikacin) for the treatment of lung infections are also available on the market [14–16]. However, in addition to local treatment of respiratory diseases, inhalable formulations are also approved for fast-acting insulin delivery [17, 18]. More inhalable formulations are expected to be introduced to the market, as numerous research in the field of inhalable DDS is present.

In this article, we present the review of recent approaches in the manufacturing of drug carriers dedicated to inhalation. We refer to almost a hundred papers from more than 10 years on different formulations made from polysaccharides, proteins, lipids and both natural or synthetic polymers, as well as composite ones. Moreover, we summarize the requirements for DPIs and compare them with other available solutions to display current trends in DPI research. Furthermore, we conclude the most recent difficulties faced in the field and forecast future trends to solve them. Thus, our review aimed to compare the pros and cons, popular manufacturing methods and current trends in the use of pulmonary DDS. We hope that our work will be helpful in determining the material in the early stages of obtaining new formulations for inhalation.

## Compared different types of inhalers

Throughout the years, many systems to deliver drugs by inhalation have been invented. The choice of devices used for this purpose often plays a critical role in the management of obstructive lung diseases. One of the most important features of an inhalation therapy device is to ensure high drug deposition in the infected area. The delivery of constant and precise doses has a great influence on both efficacy and safety. An optimal device should maintain the same performance under different conditions of use, e.g. when the inspiratory flow generated by the patient decreases. In addition, it should be able to protect the medication from environmental conditions such as temperature and humidity. Patient convenience when using the device is another important quality factor that helps it be properly used, leading to more successful therapy. Other desirable properties include low cost and environmental sustainability. Nowadays, four different types of inhalers are used: nebulizers (air jet nebulizer, ultrasonic nebulizer and vibrating mesh nebulizer), DPIs, pMDIs and soft mist inhalers [19]. Table 1 compared all types of inhalers.

**Table 1.** Comparison of different types of devices that deliver drugs to the lungs

	Advantages	Limitations	Characteristic	Examples	References
Pressurized metered dose inhaler (pMDI)	Portable Small Cost-effective Rapid administration times Sterility and prevention of backflow Protection of the drug from light, oxygen and water Patient familiarity	Requiring coordination between actuation of the device and inhalation of the dose by the patient	Drug suspended or dissolved in propellant (with surfactant and cosolvent)	Easibreathe^®^ (beclomethasone dipropionate, salbutamol)	[20–22]
Dry powder inhaler (DPI)	Small Portable Not require coordination of inhaler actuation with inhalation Deliver a relatively high dose	Sufficient inspiratory flow needed Require a pre-inhalation dose-loading step	Drug blend in lactose, drug alone, drug/excipient particles	Tobi^®^ Podhaler (tobramycin) Colobreathe^®^ (colistin)	[10, 20, 23]
Soft Mist Inhaler (SMI)	Portable Compact Multidose The relatively long generation time of the aerosol cloud facilitates coordination of inhalation and actuation. No propellants	The metered volume of 15 μl limits the dose-delivery capacity of the marketed design to drugs with the adequate solubility with respect to the required dose. The administration of tiotropium via Respimat^®^ may not be used in patients with pre-existing cardiovascular comorbidities	Aqueous solution or suspension	Respimat^®^ (ipratropium bromide, olodaterol, salbutamol, tiotropium bromide)	[20]
Air jet nebulizer		High amount of drug wastage Not all drug formulations may be appropriate	Generate aerosols from the liquid medicament using a source of compressed gas	Medix^®^ Pari LC Plus^®^	[22, 24, 25]
Ultrasonic nebulizer	Large volume of solution can be aerosolized in a relatively short period of time	Oscillation of the piezoelectric crystal results in the production of heat	Aerosol creation is based on the vibrations of a piezoelectric crystal that generate high frequency sound waves	Aerosonic^®^ Sonix 2000 systems^®^	[22, 25, 26]
Vibrating mesh nebulizer	Quiet Portable Easy to use Self-contained power source Particle size optimized for specific medications More efficient when compared to other nebulizers	Expensive Hard to clean Medication dosage requires adjustment Incompatible with viscous liquids or liquids that crystallize on drying	Use oscillation and a mesh membrane to induce droplet production through cavitation and wave formation in the liquid below the mesh	Pari eFlow^®^	[22, 27–29]

**Figure 1.**
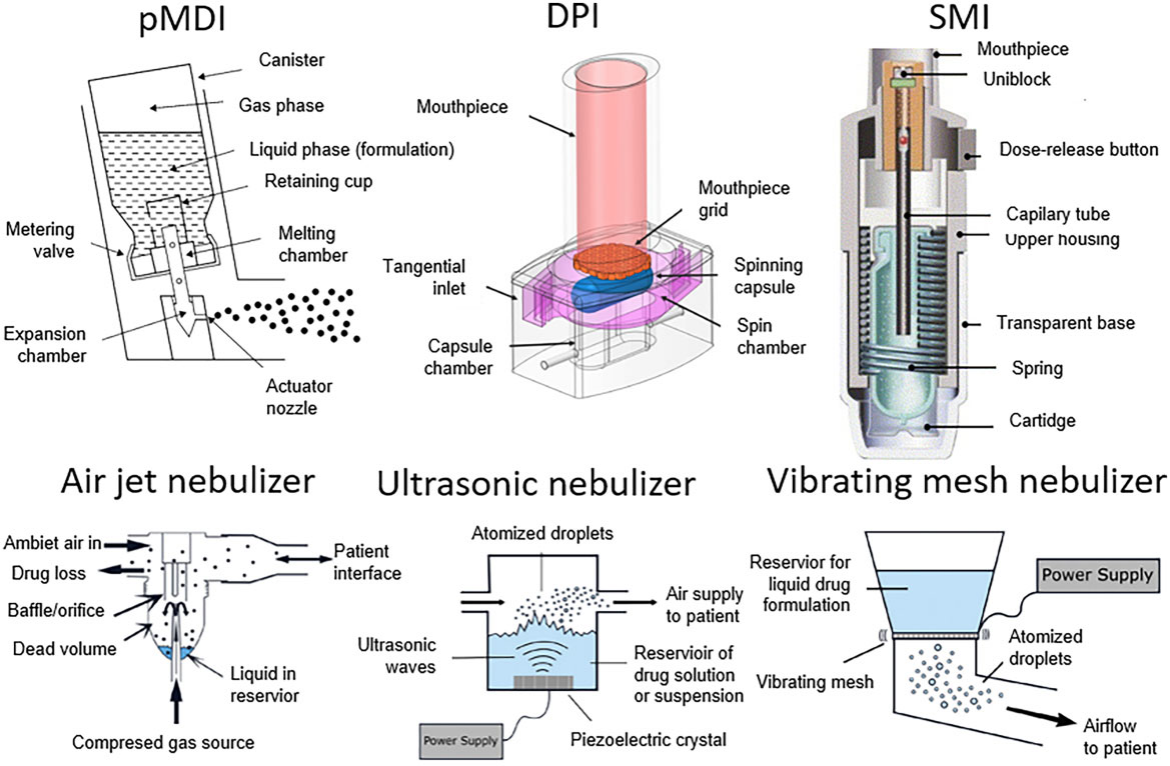
Construction and operation of inhaler devices: pressurized metered dose inhaler (pMDI) [30], dry powder inhaler (DPI) [31], soft mist inhaler (SMI) [32], air jet nebulizer, ultrasonic nebulizer and vibrating mesh nebulizer [33]. All the pictures adapted with permission.

The main idea of all the devices is similar—it is to obtain an aerosol of either liquid (e.g. nebulizers) or solid-state particles (e.g. DPIs). Both formulations may be delivered to all parts of the lungs if the proper aerodynamic diameter is ensured. The specifications of the systems and their final clinical performance differ in many aspects. The construction of different devices used to deliver drugs directly to the lungs is shown in Fig. 1.

One of the greatest advantages of DPI is the ability to deliver relatively high doses of the formulation (i.e. a pure drug or a drug encapsulated within a carrier) [23]. Akkerman-Nijland *et al*. [34] compared the eradication of *Pseudomonas aeruginosa* from the lungs of cystic fibrosis patients, delivering Tobr by nebulization and by DPI device. The results showed total bacteria eradication of 87.5% and 50% for DPI and nebulization, respectively. The results may indicate that DPI is a more effective device; however, the authors were far from a firm conclusion, as the size of the population was relatively small. On the other hand, Ishizuka *et al*. [35] compared inhalable influenza therapy with laninamivir and obtained slightly better results for nebulization than for DPI.

The disadvantage of DPIs and other solid-state formulations, which is often mentioned in the literature, is the change in the efficacy of the delivery in various breathing patterns. Sufficient inhalation velocity allows disaggregation of the powder and deep lung penetration, but such a strong inhalation may not be available in the case of young children or people with severe lung obstruction, leading to the nebulizers that provide a single dose over many breaths and through tidal breathing [28, 36].

In 2020, Terry and Dhand [37] published an extensive review on the comparison of inhalers and nebulizers for patients with stable COPD that was an update of the other review from 2005 by Dolovich *et al*. [38], claiming that there is no difference in the efficacy of therapy between both methods. The updated review concludes that nebulizers may be more effective in treatment, as COPD patients may have problems with not only inhalation, but also other physical or cognitive impairments that could limit the therapeutic influence of inhalation treatment. However, the authors did not draw a strong conclusion about the superiority of the nebulizer, but suggested that a complex study of the two methods is needed. Some other recent studies express a similar belief. Craddock *et al*. [39] noticed that patients treated with nebulizers improved their CAT score (COPD Assessment Test) more than those treated with DPI or pMDI. Leaker *et al*. [40] obtained slightly better results with glycopyrronium bromide administration by nebulization in COPD patients than with DPI administration, while Ohar *et al*. [41] showed that nebulization administration is less influenced by breathing conditions. On the other hand, Mahler *et al*. [42] did not obtain significant differences between the nebulizer and DPI in FEV_1_ (forced expiratory volume) or FVC (forced vital capacity). Baveja *et al*. [43] also did not find significant differences in both therapies using another drug. In addition, Akkerman-Nijland *et al*. [44] provided a study that showed not only that there is no clinical difference between colistin nebulization and DPI delivery in patients with cystic fibrosis, but also that DPI was more patient-friendly and was more willingly chosen.

In summary, reports can be found throughout the literature showing a slight superiority of either nebulizers or DPIs. However, they are far from making the strong statement that only one has a future perspective. The general message of the articles cited above is that the form of treatment should be tailored to each patient individually and that it is very important to make sure that the chosen device is used correctly. The responsibility for the choice should lie with healthcare providers, as most patients do not concern themselves with the influence of proper use of their device on the efficacy of the treatment [45]. From a scientific point of view, both drug delivery methods will be further developed to overcome their limitations and thus obtain better clinical results.

## Requirements for microparticles as dry powders for inhalation

Although pulmonary DDSs have many advantages, the effectiveness of treatment is conditioned by several factors, in particular: (i) the aerosol deposition site in the respiratory tract which is influenced by the physical properties of dry powder, (ii) the inhalation conditions and (iii) the condition of the patient’s respiratory airways. To succeed in therapy with DPI, it is therefore necessary to provide a formulation of the right particle size, shape, hygroscopicity, charge, etc., to overcome limitations connected with fast mucociliary clearance and airway geometry. Furthermore, the patient’s condition could be crucial because factors such as age, sex or the nature and severity of lung obstruction are highly influential in respiratory rhythm, inspiratory flow, volume of inspiration, breathing break at the end of inspiration and hand-blown coordination [25]. For this reason, it is very important to understand the whole process of the fate of the formulations on the respiratory track to be able to prepare the best solutions in the future.

After inhalation, microparticles (MPs) may be exhaled or deposited in the respiratory tract. If and where MPs will remain in upper or lower airways is influenced by the manufacturing method, their physicochemical properties and the other factors mentioned above [46]. The two parameters that characterize drug carriers are encapsulation efficiency (EE) and loading efficiency (LE) [47]. The EE determines how much of the drug used for the manufacturing of the formulation has been encapsulated, while the LE shows how much of the formulation’s mass consists of the drug itself. It is important to maximize the EE, by optimizing the manufacturing process as it allows us to obtain the particles in a repeatable manner and minimize the drug losses in the manufacturing process. EE is calculated using equation [7]:

(1) $$\mathrm{EE}=\;\frac{\mathrm{weight}\;\mathrm{of}\;\mathrm{encapsulated}\;\mathrm{drug}\;\mathrm{in}\;\mathrm{microparticles}}{\mathrm{initial}\;\mathrm{weight}\;\mathrm{of}\;\mathrm{drug}}\cdot 100\%$$

While LE is expressed by equation (2) [7]:

(2) $$\mathrm{LE}=\;\frac{\mathrm{weight}\;\mathrm{of}\;\mathrm{drug}\;\mathrm{in}\;\mathrm{microparticles}}{\mathrm{weight}\;\mathrm{of}\;\mathrm{microparticles}}\cdot 100\%$$

**Figure 2.**
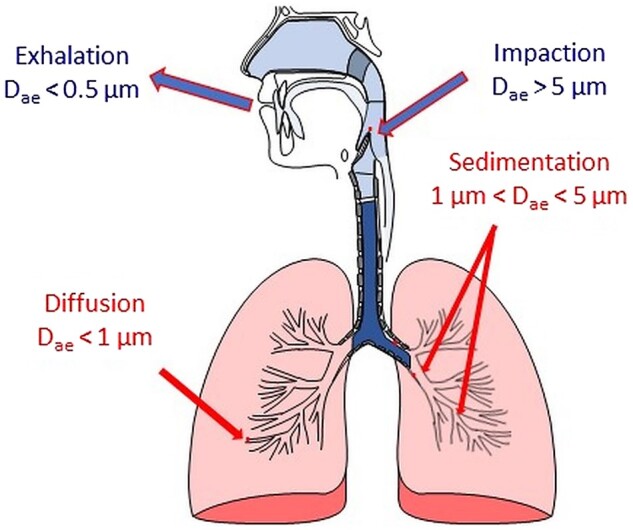
Areas and mechanisms of MPs deposition depending on their aerodynamic diameter (*D*_ae_).

The main parameter of MPs when it comes to deposition in the respiratory track is aerodynamic diameter (*D*_ae_). *D*_ae_ of the particle is its geometric diameter related to the speed of flight. *D*_ae_ may therefore be different from the geometric diameter, especially if the particles have some specific features, e.g. are highly porous. If MPs have *D*_ae_ > 5 µm, they are deposited in the upper respiratory tract using an impaction mechanism, while MPs with *D*_ae_ from 1 µm to 5 µm would remain in the bronchi and in the alveolar region to the sedimentation mechanism. MPs smaller than 1 µm, on the other hand, can diffuse into the alveoli using Brownian movements [25]. MPs with *D*_ae_ < 0.5 µm are easily exhaled. The areas and mechanisms of depositions of MPs depending on *D*_ae_ are presented in Fig. 2.

The deposition of the MPs is not only related to *D*_ae_. Their density also plays an important role. The probability of MP deposition in the lower respiratory tract is inversely proportional to the density of MPs [48]. The ideal bulk density for MPs should be <0.4 g/cm^3^. A desirable feature to obtain may be the irregular shape and porosity. Such MPs have a smaller density and consequently smaller *D*_ae_ compared to their physical size. Additionally, an irregular surface prevents the formation of agglomerates [49].

The other three aerodynamic parameters connected to *D*_ae_ are the mass median aerodynamic diameter (MMAD), geometric standard deviation (GSD) and fine particle fraction (FPF). MMAD divides MPs into two halves: 50% of MPs have *D*_ae_ lower than MMAD, while the other 50% of MPs have *D*_ae_ greater than MMAD [25]. GSD in turn is expressed as a square root of the diameter at the 84th centile divided by the diameter at the 16th centile (Equation 3). That is because in the log-normal distribution characteristic for the particle size distribution, 68% of the particles should fit between MMAD × GSD and MMAD/GSD.

(3) $$\mathrm{GSD}=\sqrt{\frac{d_{84}}{d_{16}}}$$

If MPs have a GSD value equal to 1, they are monodispersed, whereas GSD > 1.2 MPs are heterodispersed [50, 51]. Finally, the FPF is defined as the percentage of particles that have an aerodynamic diameter of <5 µm. The FPF is calculated using equation [25]:

(4) $$\mathrm{FPF}=\frac{\mathrm{Fine}\;\mathrm{particle}\;\mathrm{dose}\;(\mathrm{Dae}\;<5\;\mu m)}{\mathrm{Delivered}\;\mathrm{dose}}\cdot 100\%$$

where the fine particulate dose is the mass of particles with *D*_ae_ <5 μm and the delivered dose is the total mass of the drug administered to the device from the mouthpiece of the inhaler [25].

Two other parameters also characterize the MPs for inhalation, which are less often described, but could be found in the literature. These are the median diameter of the size distribution (D_V_50) and the emitted dose (ED). The value of D_V_50 indicates that the particle size in micrometers is half of the total amount of dry powder delivered from the device during inhalation. ED is the amount of drug that leaves the device and is expressed as a percentage [49].

As can be seen, there are many factors related to the deposition of MPs in the lungs, but it is not the end of the requirements. After MPs successfully reach the respiratory tract, they are exposed to a specific environment leading to erosion, dissolution, release of the drug, absorption to the blood or the clearance process (mucociliary mechanism, alveolar macrophages, enzymatic degradation) [48].

The mucus layer acts as a physical barrier and protects the tissues laying below. Mucus is composed of water (90% or more) and mucin glycoproteins (1–5%). Additionally, electrolytes, cells, or cell debris, lipids, soluble proteins, enzymes and various immune factors are present. Mucin fibers are cross-linked, with variable porosity, and have diameters of 3–10 nm, so the delivered MPs are trapped in the mucus and washed away unless they have certain properties. Successful mucus penetration is related to a sufficiently small and hydrophilic surface and neutral charge. Mucus is hydrophilic. As a result, hydrophobic MPs may be entrapped within the mucus. Positively charged MPs interact with negatively charged sialic acid residues of mucins, whereas negatively charged MPs are repulsed [52, 53]. If MPs cannot penetrate the mucus, they are removed using the mucociliary mechanism in which the mucus cooperates with the cilia to eradicate MPs from the lungs, using its rhythmical movement, which eventually causes the mucus to be expelled from the lungs [54].

Another important mechanism of potential clearance mechanism is macrophage phagocytosis. The task of macrophages is to absorb and digest insoluble MPs and deposit them in alveoli. For this reason, MPs are often not able to stay long enough in the targeted area to release the drug, even if they reach it properly. The phagocytosis of MPs depends on their geometric diameter. MPs with geometric diameter ranges of 0.5–3 µm are also easily phagocytosed, which is, unfortunately, the right size for inhalation, causing a serious problem in the development of inhalable DDSs. Therefore, possible surface treatments are tested to allow the MPs not to be removed too early [54]. The effects of phagocytosis also depend on the shapes, charge, porosity, solubility, hydrophobicity and surface modifications of MPs. Research shows that spherical MPs are easily phagocytosed; therefore, MPs in other shapes (elongated, rod and filament) can avoid macrophages. The important role also plays the Zeta potential of MPs. MPs that have some charge are easier to phagocytose than MPs with a neutral charge. Insoluble MPs can adsorb lung surfactant proteins that assist in phagocytosis by alveolar macrophages. The high solubility or hydrophilicity of MPs reduces the chances of their recognition by macrophages [55]. Furthermore, macrophages could stimulate the immune response to inhaled MPs, generating excessive inflammation [54]. However, sometimes it is necessary for MPs to be highly absorbed by macrophages (e.g. in the treatment of tuberculosis).

## MPs based on polysaccharides

**Figure 3.**
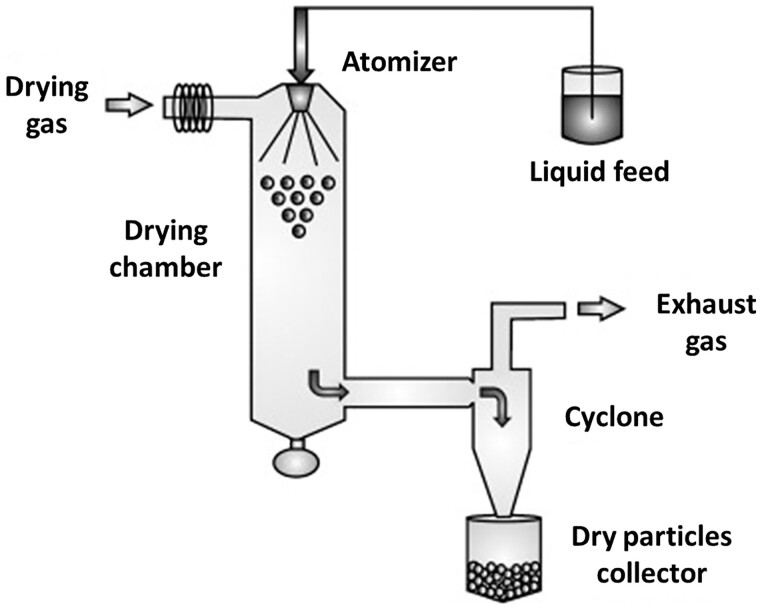
Diagram of the equipment and the process of conventional spray drying [59]. The picture adapted with permission.

Polysaccharides are natural, biodegradable, non-toxic and functional biomacromolecules that are composed of a large number of monosaccharide units linked by glycosidic bonds [56]. Polysaccharides are easily conjugated or complexed with other macromolecules. Moreover, polysaccharides can form cross-linked networks with the ability to water swelling, allowing controlled release of the drug in contact with body fluids. The most popular method to obtain MPs from polysaccharides is spray drying. This well-established technique for generating dry powdered products usually produces spherical particles as a result of the liquid surface tension upon drying of the atomized droplets. However, by adjusting the conditions under which spray drying is carried out, the particles can have different shapes, densities, geometric diameters and surface properties [57]. In Fig. 3, the equipment and process of manufacturing the MPs using the spray-drying method is presented. Spray drying consists of feeding a liquid stream (solution, suspension or emulsion) that is continuously divided into fine droplets (atomization) and transferred into a chamber (drying chamber). In the drying chamber, the droplets encounter a hot gas, and by an evaporative cooling process, they are converted into solid particles. These particles are then separated from the wet drying gas by a suitable separation system, most commonly a cyclone or filter bag [58].

Polysaccharides used in pulmonary drug delivery among others are chitosan, hyaluronic acid, locust bean gum, fucoidan, chondroitin sulfate, alginate, carrageenan and dextran [57]. Examples of polysaccharides, their applications and their properties are shown in Table 2.

**Table 2.** Applications, manufacturing method and properties of polysaccharides used in pulmonary drug delivery

Polysaccharide	API	Manufacturing method	MPs properties	Aerodynamic properties	References
Chitosan	Budesonide (asthma)	Spray drying	**Morphology:** irregular wrinkled structure	**MMAD:** 3.41–3.70 µm (depending on molecular weight of chitosan) **FPF:** 43–47% (depending on molecular weight of chitosan)	Zhang *et al*. (2018) [61]
Locust bean gum	Rifabutin (RFB) and isoniazid (INH) (tuberculosis)	Spray drying	**Morphology:** irregular-shaped MPs with convoluted surface **EE:** 102 ± 1% (RFB) and 94 ± 3% (INH) **LE:** 4.4 ± 0.1% (RFB) and 8.2 ± 0.3% (IHN)	**MMAD:** 5.8 ± 0.3 µm (RFB) and 6.2 ± 0.6 µm (INH) **FPF:** 38.1 ± 1.8% (RFB) and 38.0 ± 1.6% (INH)	Grenha *et al*. (2020) [66]
Hyaluronic acid	Salbutamol sulfate (asthma, COPD)	Spray drying	**Size (D_50_):** 4.9 ± 4.0 µm **Morphology:** spherical shape, dimpled and corrugated surface **EE:** 95.5 ± 1.4% **LE:** 22.0 ± 0.3%	**ED:** 89.6 ± 4.7% **FPF:** 32.8 ± 0.4% **MMAD:** 4.2 ± 0.1 µm **GSD:** 2.1 ± 0.1	Li *et al*. (2017) [62]
Fucoidan	Rifabutin (RFB) and isoniazid (INH) (tuberculosis)	Spray drying	**Morphology:** irregular and acquired corrugated surfaces	**D_V_50:** 2.77 ± 0.03 µm **MMAD:** 3.64 ± 0.32 µm (RFB) and 3.90 ± 0.01 µm (INH) **ED:** 1.10 ± 0.02 mg (RFB) and 1.64 ± 0.23 mg (INH) **FPD:** 0.53 ± 0.01 mg (RFB) and 0.82 ± 0.02 mg (INH) **FPF:** 38.1 ± 1.8% (RFB) and 38.0 ± 1.6% (INH)	Cunha *et al*. (2018) [65]
Phytoglycogen	Rifampicin (tuberculosis)	Spray drying	**Size (D_50_):** 2.86–10.19 µm (depending on concentration of ethanol used as solvent) **Morphology:** wrinkled shapes **EE:** 94.45–105.95% (depending on concentration of ethanol used as solvent)	**FPF:** 20–55% (depending on concentration of ethanol used as solvent)	Tse *et al*. (2021) [64]
Chondroitin sulfate	Rifabutin (RFB) and isoniazid (INH) (tuberculosis)	Spray drying (ethanol used as solvent)	**Morphology:** wrinkled and corrugated surface **EE:** 59.0 ± 6.9% (RFB) and 94.9 ± 5.7% (INH) **LE:** 2.6 ± 0.3% (RFB) and 8.2 ± 0.5% (IHN)	**D_V_50:** 4.1 ± 0.1 µm **MMAD:** 3.9 ± 0.1 µm (RFB) and 3.8 ± 0.1 µm (INH) **ED:** 90.9 ± 1.0% **FPD:** 1.5 ± 0.1 mg (RFB) and 3.1 ± 0 mg (INH) **FPF:** 42.6 ± 1.7% (RFB) and 43.7 ± 2.4% (INH) **GSD:** 2.0 ± 0.1 (RFB) and 1.9 ± 0.1 µm	Rodrigues *et al*. (2020) [63]

MP, microparticles; EE, encapsulation efficiency; LE, loading efficiency; MMAD, mass median aerodynamic diameter; ED, emitted dose; FPD, fine particle dose; GSD, geometric standard deviation; FPF, fine particle fraction.

**Figure 4.**
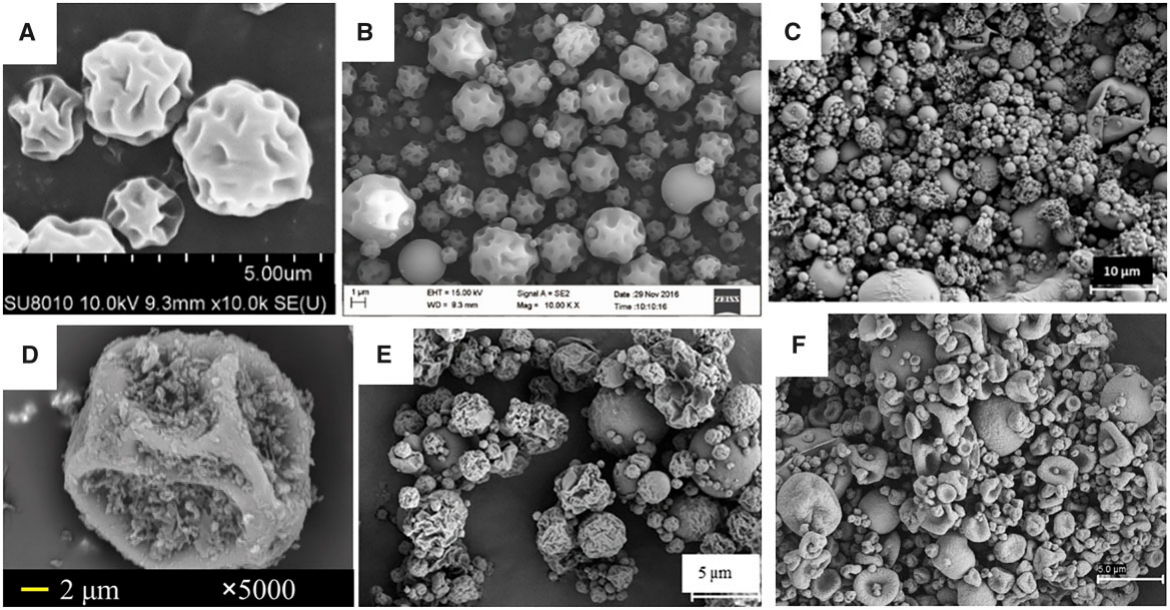
SEM images of MPs based on different polysaccharides manufactured using spray-drying method: (**A**) Budesonide-loaded chitosan swellable MPs [61], (**B**) salbutamol-loaded hyaluronic acid MPs [62], (**C**) chondroitin sulfate/isoniazid/rifabutin MPs produced with water–ethanol as solvent (mass ratio of 10/1/0.5) [63], (**D**) rifampicin and phytoglycogen (1/5, w/w) prepared in solvents containing 50% ethanol by volume [64], (**E**) fucoidan/isoniazid/rifabutin MPs (mass ratios of 10/1/0.5) [65] and (**F**) locust bean gum/isoniazid/rifabutin MPs (mass ratio of 10/1/0.5) [66]. All the pictures adapted with permission.

Chitosan is one of the most widely used polysaccharides for pulmonary drug delivery [57]. Chitosan is obtained by N-deacetylation of chitin; the degree of deacetylation of chitosan ranges from 40 to 98%, with its molecular weight starting at 50 kDa and reaching up to 200 kDa. The deacetylation of chitin provides chitosan with free amino functional groups, which, in combination with naturally occurring OH groups, leads to flexibility in its modification of it for specific pharmaceutical or medical purposes. Changing the molecular weight and degree of deacetylation allow MPs to be manufactured with the intended sizes and Zeta potentials. Chitosan has also mucoadhesive, anticancer, antibacterial, antifungal, antioxidant, anti-inflammatory and even antidiabetic properties [60]. Zhang *et al*. [61] manufactured chitosan-based swellable MPs loaded with budesonide using the spray-drying method (Fig. 4A). They presented the difference between MPs manufactured with chitosan with molecular weights of 50 kDa (SM50) and 100 kDa (SM200). The results showed that SM50 had a smaller MMAD (3.41 ± 0.26 µm) and a higher FPF (47 ± 5%) than SM200 (3.70 ± 0.14 µm; 43 ± 2%). Furthermore, *in vitro* release of budesonide after 12 h was faster for SM50 (almost 90%) than for SM200 (∼70%). The release of the drug was also evaluated *in vivo* in mice with asthmatic allergies. The single dose of SM50 and SM200 caused a delay in drug release and, as a result, the therapeutic effect had lasted 12 h for SM50 and 18 h for SM200. SM200, which showed the most retarded drug release behavior *in vitro*, resulted in the best therapeutic outcome after one single administration of budesonide.

The next polysaccharide widely used for manufacturing pulmonary formulations is hyaluronic acid—a linear polymer composed of disaccharide monomers. It is negatively charged and is easily biodegradable by native enzymes. Hyaluronic acid is mucoadhesive due to the hydrogen bonding between the carboxyl groups of the glucuronic acid residues and glycoproteins present in the mucus. Furthermore, hyaluronic acid suppresses alveolar macrophage phagocytosis, prolonging the presence of drug carriers in the respiratory track [57, 62, 63]. Li *et al*. [62] manufactured MPs based on hyaluronic acid loaded with salbutamol sulfate (SAS) using the spray-drying method (Fig. 4B). In the study, the comparison between SAS dry powder (SAS/DP) and dry powder of SAS loaded HA MPs (SAS-HA/MP) was presented. The results showed that SAS-HA/MP possessed slightly higher ED and lower FPF compared to SAS/DP, whereas there was no statistically significant difference between the two formulations. The *in vivo* test on rats showed that the retained amount of SAS locally distributed in the lungs was more than three times higher in the case of SAS encapsulated in hyaluronic acid MP than in the case of SAS without HA for the entire time. In addition, SAS retention time of SAS was also significantly prolonged from 2 to 8 h with the aid of hyaluronic acid.

In addition to the two mentioned, there are also less popular polysaccharides used to obtain MPs. In 2020, Rodrigues *et al*. [63] manufactured MPs based on chondroitin sulfate loaded with isoniazid (INH) and rifabutin (RFB) mass ratio of 10/1/0.5. Chondroitin sulfate is a natural polymer commonly found in proteoglycans in several tissues, including the lungs. The MPs obtained were spherical, wrinkled and corrugated (Fig. 4C). The dose emitted by the inhaler was very high, reaching 90% and its FPF of 34–44% was determined. A similar volume of FPF (38.9 ± 5.58%) had MPs based on phytoglycogen manufactured by Tse *et al*. [64]. Phytoglycogen exists as natural hyperbranched starch-like dendritic nanoparticles (NPs) that are biosynthesized mainly in sugary mutant grains that lack debranching enzymes [67]. The MPs obtained based on phytoglycogen were in the shape of small flakes attached to the uneven surfaces of particles with wrinkled structures (Fig. 4D). Most of the flakes had diameters <2 μm, whereas large and wrinkled particles ranged from 10 to 15 μm in diameter [64]. The next example is the MPs based on fucoidan manufactured by Cunha *et al*. [65]. Fucoidan is a water-soluble polysaccharide that consists mainly of l-fucose and sulfate groups, in addition to other components such as mannose, glucose, xylose and glucuronic acid [68]. The MPs based on fucoidan were loaded with INH and RFB (mass ratios of 10/1/0.5). The MPs obtained were irregular and acquired corrugated surface (Fig. 4E). Their aerodynamic properties were very promising: MMAD was between 3.6 µm and 3.9 µm, FPF was around 50% and ED was around 85% [65]. Grenha *et al*. [66] manufactured MPs of locust bean gum loaded with INH and RFB. Locust bean gum is produced from the seed of the locust bean tree (carob tree), *Ceratonia siliqua* [69]. MPs obtained on this material generally revealed an irregular shape with a convoluted surface (Fig. 4F). The MMAD determined for the MPs was around 6 µm and the FPF was 38%.

## MPs based on proteins

Proteins are made from a long chain of amino acids connected to each other with a covalent peptide bond. There are 20 types of amino acids in proteins with different chemical structures and properties. Each type of protein has a unique sequence of amino acids that are exactly the same from one molecule to another. Many thousands of different proteins are known, and each of them has its own particular amino acid sequence [70].

Protein-based DDSs have main advantages: biodegradation, stability and easy control of particle size [71]. MPs can be made of various proteins commonly produced by living organisms, e.g. collagen and its hydrolyzed form, namely gelatin, fibrin, silk fibroin, keratin, albumin or sericin [72]. However, proteins as drug carriers also have some limitations. They are very sensitive to temperature and shear stress during manufacturing processes (e.g. freeze-drying, spray drying), which can lead to the protein degradation. Inhaled protein may undergo various degradation mechanisms during production, processing and/or storage. These degradation pathways may be physical (denaturation and noncovalent aggregation) or chemical (mainly covalent aggregation, deamidation, oxidation and/or glycation) [73]. This could be a reason why there are fewer research projects dedicated to protein-based inhalable formulations compared to other types of MPs. Examples of protein-based MPs used in pulmonary drug delivery are presented in Table 3.

**Table 3.** Applications, manufacturing method and properties of MPs based on proteins used in pulmonary drug delivery

Protein	API	Manufacturing method	MPs properties	Aerodynamic properties	References
Silk fibroin	Cisplatin (lung cancer)	Spray freeze-drying	**Size (D_50_):** 5.20 ± 0.69 μm	**FPF:** 62.25%	Kim *et al*. (2015) [75]
Silk fibroin	Ciprofloxacin (non-cystic fibrosis bronchiectasis)	Spray drying	**Size (D_50_):** 2.95 ± 0.05–4.55 ± 0.10 **Morphology:** spherical shape with shrinkage surface (depending on drug loading)	**MMAD:** 3.75 ± 0.03–4.66 ± 0.17 µm **ED:** 94.49 ± 0.84–98.10 ± 1.27% **FPF:** 36.77 ± 3.86–45.04 ± 0.84% **GSD:** 1.66 ± 0.10–4.23 ± 0.16 (depending on drug loading)	Liu *et al*. (2019) [76]
Polylysine + dextrin	Indomethacin (rheumatoid arthritis)	Spray-drying	**Size (D_50_):** 5.99 ± 0.27 µm, **Morphology:** non-porous **LE:** 20 ± 1%	***D*_ae_:** 4.79 µm, **FPF:** 51.55 ± 0.37%	Ceschan *et al*. (2015) [77]

MPs, microparticles; MMAD, mass median aerodynamic diameter; ED, emitted dose; GSD, geometric standard deviation; FPF, fine particle fraction.

Among existing proteins, the only one that currently appears to be investigated as a potential inhalable DDS is silk fibroin that is commonly produced by silkworms [71]. Fibroin is insoluble in water and is a nontoxic, hydrophobic and histocompatible glycoprotein [72]. Moreover, silk-based DDSs can promote drug delivery through the mucus layer by increasing particle residence time and, therefore, improving drug efficacy [74]. It was first used by Kim *et al*. in 2015 [75] to manufacture silk fibroin-based MPs for pulmonary drug delivery. The MPs were produced using the spray freeze-drying or spray-drying method. Three batches of MPs were obtained—empty, containing cisplatin, and cross-linked, containing cisplatin. The results showed that the MPs had high aerosolization efficiency. In 2019, Liu *et al*. [76] also developed MPs based on silk fibroin loaded with ciprofloxacin (Cip) at different concentrations. The MPs obtained were spherical in shape and rough on the surface. The results showed that the MPs had a high ED in the range of 94–98% and a FPF in the range of 36–45%.

Natural proteins and saccharides have a relatively high number of reactive groups in their structure. This could be used to synthesize a polymer from them that mimics natural polymers based on for example peptide bond. In 2015, Ceschan *et al*. [77] published a paper on the encapsulation of indomethacin, an anti-inflammatory drug used in rheumatoid arthritis, in a mixture of two polymers: polylysine and dextrin. Lysine is an amino acid with two amino groups, so after polymerization, there is still one free first-order amino group. Indomethacin has carboxyl groups in its structure, which create the possibility of chemical bonding of the drug to the polymer structure. Formulations of various substrate ratios were prepared by the classic spray-drying method without the use of an organic solvent and with relatively high LEs.

## MPs based on synthetic polymers

Poly(lactide-*co*-glycolide) (PLGA) is one of the most widely investigated synthetic degradable polymers in the field of biomaterials. Among many possible applications, drug delivery to different tissues seems to be highly promising. This is because PLGA is a copolymer that could be obtained from lactic acid (or lactide) and glycolic acid (or glycolide) in various ratios and molecular weights that influence the properties important for drug release, e.g. degradation kinetics [78]. Also, there are plenty of PLGA carriers manufacturing methods that allow for the creation of the particles in a diameter range between ca. 100 nm to several hundred µm. The most common manufacturing methods are single or double emulsification, which is easy and convenient to encapsulate hydrophilic or lipophilic drugs. The method is, however, problematic to convert to large-scale manufacturing. The other methods are spray drying, a popular, scalable method that requires a higher temperature that could degrade some drugs, and microfluidic devices, an upgraded version of the emulsification method that allows the obtaining MPs of the homogeneous size distribution with great reproducibility [79–81].

**Figure 5.**
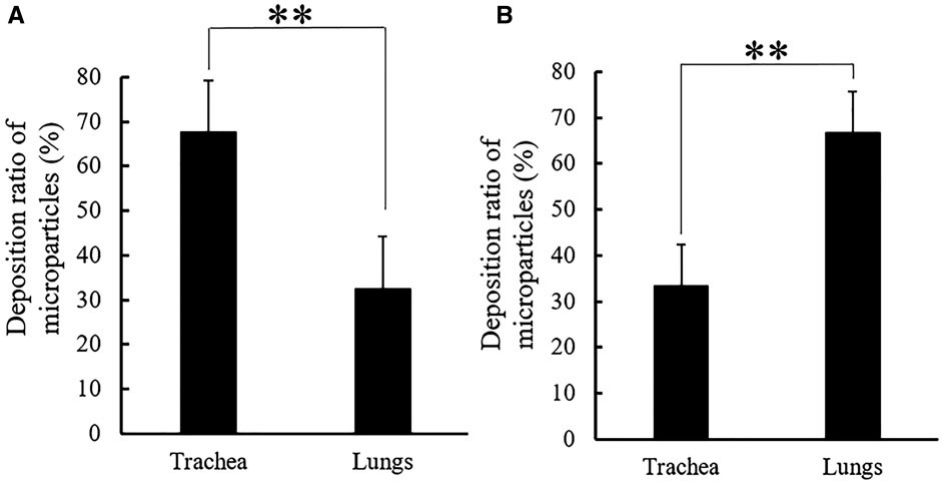
Comparison of the deposition *in vivo* of PLGA MPs obtained by spray-drying in solvent with DCM:methanol ratio 100:0 (**A**) and 70:30 (**B**) (**p < 0.01, Tukey’s test) [85]. The picture adapted with permission.

**Figure 6.**
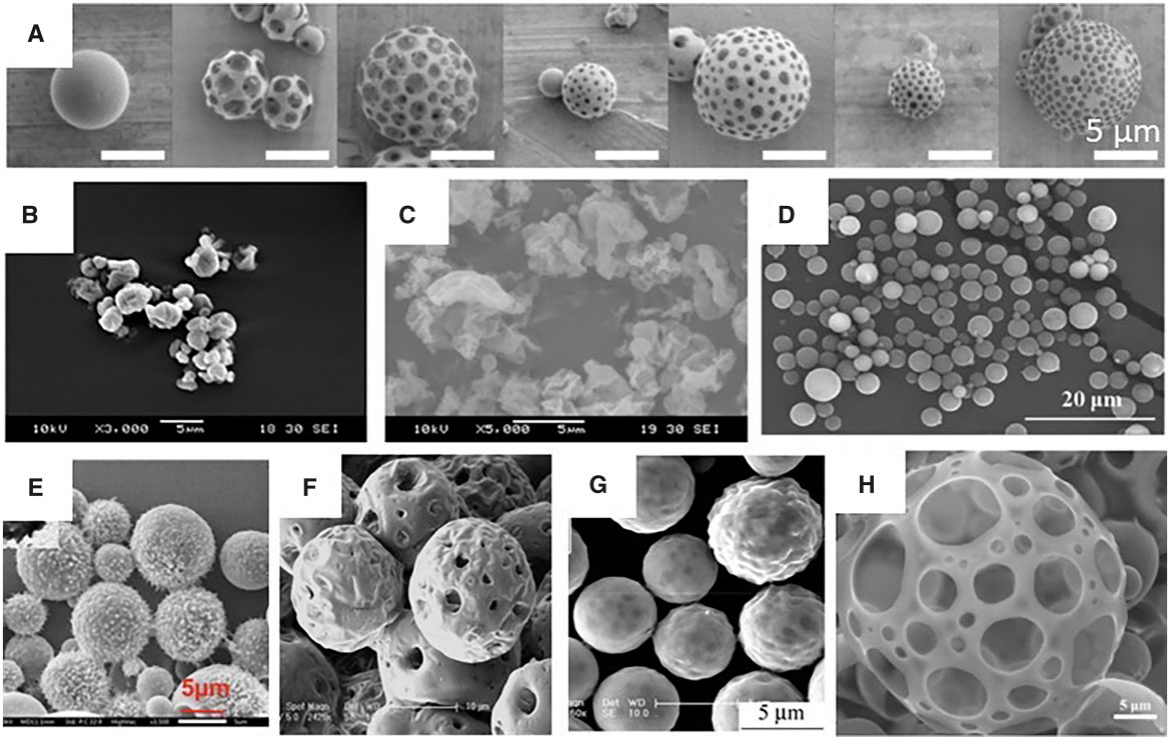
Various PLGA MPs obtained in different conditions and for different purposes. (**A**) Non-porous and porous MPs with various morphologies due to the changing homogenization rate and surfactants—single emulsification [82]; (**B**) spray-dried MPs with the mixture of DCM: methanol 70:30 [85]; (**C**) spray-dried MPs modified with 0.2% of leucine for non-spherical morphology for increased FPF [88]; (**D**) surface-modified with PEG-2000 MPs obtained by premix membrane double emulsification to avoid macrophage uptake [87]; (**E**) *N*-acetyl cysteine surface-modified MPs obtained by double emulsification for better mucus presentation [89]; (**F**) porous by the use of ammonium bicarbonate MPs obtained by double emulsification [90] and porous MPs from double emulsification; (**G**) with internal pores, loaded with doxorubicin [91]; and (**H**) with external pores, loaded with artesunate [92]. All the pictures adapted with permission.

Inhalable drug carriers should reach the side of action and remain there as long as necessary to release the therapeutic agent. Nishimura *et al*. [82] showed that porous PLGA MPs obtained by a single emulsification method are more likely to reach the lungs (bronchi, alveoli) and stay there longer while inhaled than nonporous ones. Moreover, increasing porosity accelerates drug release, e.g. BAY 41-2272, as proved by Zhang *et al*. [83]. On the other hand, most of the MPs remained in the experiment apparatus (Next Generation Impactor, NGI), although the FPF reached up to 48%. Faster drug release was also observed in porous PLGA MPs by Ni *et al*. [84] who also showed that such MPs (Fig. 6A) may escape phagocytosis. The problem of low FPF was investigated by Nii *et al*. [85]. They studied the influence of the conformation of PLGA molecules on the lung delivery of spray-dried MPs (Fig. 6B). The work showed that the aerodynamic diameters of the MPs can be improved by adding methanol to the organic solvent (dichloromethane, DCM),—FPF increased from 9.02 ± 1.56% to 40.99 ± 4.58%—and therefore led to a higher lung deposition (Fig. 5).

Another field of research dedicated to inhalable MPs is different surface modification to influence macrophage uptake. Li *et al*. [86] modified the PLGA MPs surface with phospholipids mimicking the lung environment—the lung surfactant fluid—that was either modified with poly(ethylene glycol) (PEG) or not and tested the uptake and clearance by macrophages *in vitro* and *in vivo.* The non-PEGylated phospholipid-modified PLGA MPs showed enhanced macrophage uptake compared to those of the unmodified ones, as the PEGylated ones could resist macrophage clearance for a significantly longer time. The authors concluded that the MPs surface–lung fluid interaction is crucial when it comes to the design of DDS with controlled release. The influence of PEG surface modification on mucus penetration and macrophage uptake was also investigated *in vitro* and *in vivo* by Li *et al.* [87]. The study showed that if the PEG molecules are too short (PEG 750 Da), they have no influence on MP retention in the lungs. The MPs modified with high molecular PEG, i.e. 5 or 10 kDa, showed increased mucus penetration and decreased macrophage uptake; however, the release of budesonide from the MPs was affected. The best performance was demonstrated by PEG 2 kDa (Fig. 6D), which delayed macrophage clearance, but had no negative impact on drug release kinetics.

Inhalable PLGA MPs have been analyzed as a promising treatment for tuberculosis. Drugs to be delivered were rifampicin [88–91], moxifloxacin [94], gatifloxacin [95] and novel alternatives such as all-trans-retinoic acid [96] or antibacterial peptides [89]. An interesting approach to inhalable polymeric drug carriers was modification with amino acids to obtain non-spherical MPs. Takeuchi *et al*. [88] used L-aspartic acid and different concentrations of L-leucine in spray-dried manufactured rifampicin-loaded PLGA MPs to treat tuberculosis (Fig. 6C). This strategy allowed one to increase FPF up to 6.9 times. Although the EE decreased slightly, macrophage uptake improved and the drug release rate was much faster than for typical PLGA MPs tested before [94, 95]. *N*-acetyl cysteine was also indicated to significantly improve the mucus penetration capacity of antibacterial peptide (IDR-1018) loaded PLGA MPs (Fig. 6E) [89]. Furthermore, Hirota *et al*. [93] designed a device based on the Venturi effect to deliver PLGA MPs to treat tuberculosis. The use of other synthetic polymers to this approach has been marginal in recent years. Previous studies covered the approach of, e.g. poly(lactic acid) (PLA), polycaprolactone (PCL) or hydroxyl propyl methylcellulose [96], but there is no visible trend in the latest literature to continue.

Tuberculosis is not the only disease that can be treated with inhalable polymeric MPs. This way of drug delivery is also dedicated to infections associated with chronic diseases, e.g. cystic fibrosis, COPD, asthma, idiopathic pulmonary fibrosis and other diseases associated with an increased risk of bacterial infections. Therefore, the pulmonary tract is also investigated for the delivery of antibiotics or other antibacterial agents, and polymeric MPs are one of the possible approaches.

Antibiotics commonly encapsulated in polymeric MPs are azithromycin (Azi) [97], levofloxacin [98] or Tobr [99]. To obtain antibiotic-loaded MPs, common synthetic polymers are PLGA and PCL. Gaspar *et al*. [98] analyzed levofloxacin encapsulation within PLGA MPs as a possible DDS for CF-related lung infections. The MPs were manufactured using the water–in oil–in water emulsification method with premix membrane homogenization. The authors prepared several batches of MPs obtained under different conditions. They tried to add lauric acid to the oil phase, but it was not sufficient. In fact, the drug loading was higher; however, the diameter sizes increased significantly, making the MPs not suitable for inhalation. Therefore, the formulation chosen by the authors showed an EE of 23.1% and a drug loading of 10.5 ± 1.4, which is not very high, but satisfactory. Ernst *et al*. [99] created PLGA and PEG-PLGA MPs and NPs loaded with Tobr to overcome the obstacles of mucus penetration and biofilms that are an inevitable problem when it comes to bacterial infections. MPs were obtained by double emulsification (changing the magnetic stirring to obtain MPs or NPs—6000 rpm and 24 000 rpm, respectively) with the use of ethyl acetate as an oil phase. Neither EE nor LE were high (around 2–4% and around 0.1–0.2% for EE and LE, respectively), resulting in poor minimum inhibitory concentration values against *P. aeruginosa* and *Burkholderia cepacia.* On the other hand, the tests provided on the matured biofilm showed that MPs (and NPs as well) showed superior antimicrobial activity against both strains of bacteria compared to pure Tobr in a dose that was estimated by the authors to be the highest possible encapsulated dose. The dose of pure Tobr to affect the biofilms was 1000 mg/l, which is not suitable for the treatment of patients. Here, the researchers faced one of the main problems associated with hydrophobic synthetic polymers, i.e. a very limited hydrophilic drug encapsulation capacity. However, the study presents an important outcome, showing that if the drug-carrier affinity could be enhanced, it would be a promising way to use MPs (or NPs) to treat biofilm infections.

PCL is another popular biodegradable polymer that caught the attention of scientists as a potential antibiotic carrier. Kasten *et al*. [97] used it to obtain Azi-loaded MPs by double emulsification to treat pneumonia. It is an interesting approach, as Azi is a lipophilic drug and MPs could be manufactured by single emulsification. Herein, both the drug and the polymer were dissolved in DCM. In the water-1 phase, ammonium bicarbonate and poloxamer 188 were added to decrease density and prevent macrophage uptake. The highest EE obtained was 23.07 ± 0.31% and batches obtained with a reduced amount of DCM generally showed a better result. Aerodynamic studies *in silico* indicated that these MPs may reach the alveolar region of the lungs; however, the estimated number of MPs delivered to the lungs differed when the manufacturing parameters were changed.

Treatment of bacterial infections is not only related to the delivery of antibiotics. As bacteria strains become increasingly resistant to currently used therapeutics, other approaches are widely investigated, also in the field of pulmonary delivery. These agents could be, e.g. bacteriophages—viruses that exclusively target bacteria [100], curcumin, a natural food coloring factor with anti-inflammatory properties that is also a *quorum sensing* inhibitor [101, 102], or other anti-inflammatory substances of natural origin such as *Alstonia scholaris* alkaloids in an interesting copolymer of PEG and poly(mandelic acid) [103]. The delivery of bacteriophages to the lungs by Agarwal *et al*. [100] is especially interesting. The team proved that the viruses would not lose the potential to affect bacteria due to the encapsulation process. PLGA MPs containing phages (Fig. 6F) were able to cure mice from acute pneumonia *in vivo*, showing a promising alternative to antibiotic use in light of the phenomenon of antibiotic–antibacterial resistance. In particular, the phages did not cause damage to healthy lungs. Natural anti-inflammatory compounds, e.g. curcumin, are also suitable for delivery via inhalation in polymeric MPs. Kwiecień *et al*. [102] prepared non-porous curcumin-loaded MPs from poly(sebacic anhydride)—a material with a very short degradation time to release the drug before macrophage uptake, while Hu *et al*. [101] manufactured very similar particles from PLGA—a slower degrading polymer—but with additional ammonium hydroxycarbonate added to the water phase to make them porous, so that the MP would remain in the alveoli for longer, before being uptaken by the macrophages.

Polymeric MPs are also widely investigated in the therapy of lung cancers. In this field, PLGA MPs also play an important role. Although there are many drugs used in cancer therapy, the most widely investigated is doxorubicin (DOX) alone [104, 105] or combined with other therapeutic agents, e.g. paclitaxel (PXT) [106], miR-519c (microRNA to improve intracellular concentration of chemotherapeutic drugs) [107], TRAIL ((TNF)-related apoptosis-inducing ligand) [91] or p53 (gene-therapeutic agent) [90]. The other drugs encapsulated in PLGA MPs for this approach are artesunate [92], metformin with docosahexaenoic acid (DHA) [108], oridonin [109] or disulfiram [110].

All of the investigated MPs were obtained by single or double emulsification (depending on the solubility of the drug). Although the suitable particle size for inhalation should not exceed 5 µm, in most of the presented studies, the MPs have diameters over 10 µm up to several dozen µm. In such cases, the MPs are highly porous to change their aerodynamic properties so that they can reach deeper parts of the lungs than nonporous ones of such sizes. This approach also allows MPs to remain longer in the lungs because, with this morphology, they are less likely to be absorbed by alveolar macrophages.

The general problem of chemostatic drug delivery in PLGA MPs is poor drug loading—very rarely above 10% w/w. Recently, the two approaches have been used to improve the efficacy of antitumor treatment with PLGA MPs. One is to combine PLGA with another polymer so that parameters such as for example release kinetics are more appropriate for this purpose. Li *et al*. [104] mixed PLGA with poly(cyclohexane-1,4-diyl acetone dimethylene ketal) (PCADK), a fast degrading material, to improve the antitumor effect. They found that the optimal weight ratio is 2/8 PCADK/PLGA, since the addition of PCADK indeed improved the performance of DOX-loaded MPs but at the same time increased aerodynamic diameter (2.48 ± 0.18 and 5.17 ± 0.70 for the 2/8 and 8/2 PCADK/PLGA ratios, respectively) and decreased EE (77.22 ± 4.32 and 21.42 ± 5.01 for the ratio 2/8 and 8/2 PCADK/PLGA ratios, respectively). However, by using the optimal ratio, it was possible to accelerate MP degradation—the cumulative release of DOX was at a level of 64.66% after 10 days compared to the pure PLGA MPs (46.31%). The enhanced antitumor effect was also shown *in vitro* and *in vivo*. PCADK/PLGA MPs had better antiproliferative properties in A549 lung cancer cells. In addition, they can reduce cancer expansion in a lung cancer-bearing BALB/c mouse model—the average number of tumor nodes per lung was 17, 10, 6 and 3 without treatment, DOX intravenous injection, DOX-loaded PLGA inhaled MPs and DOX-loaded PCADK/PLGA inhaled MPs, respectively.

**Figure 7.**
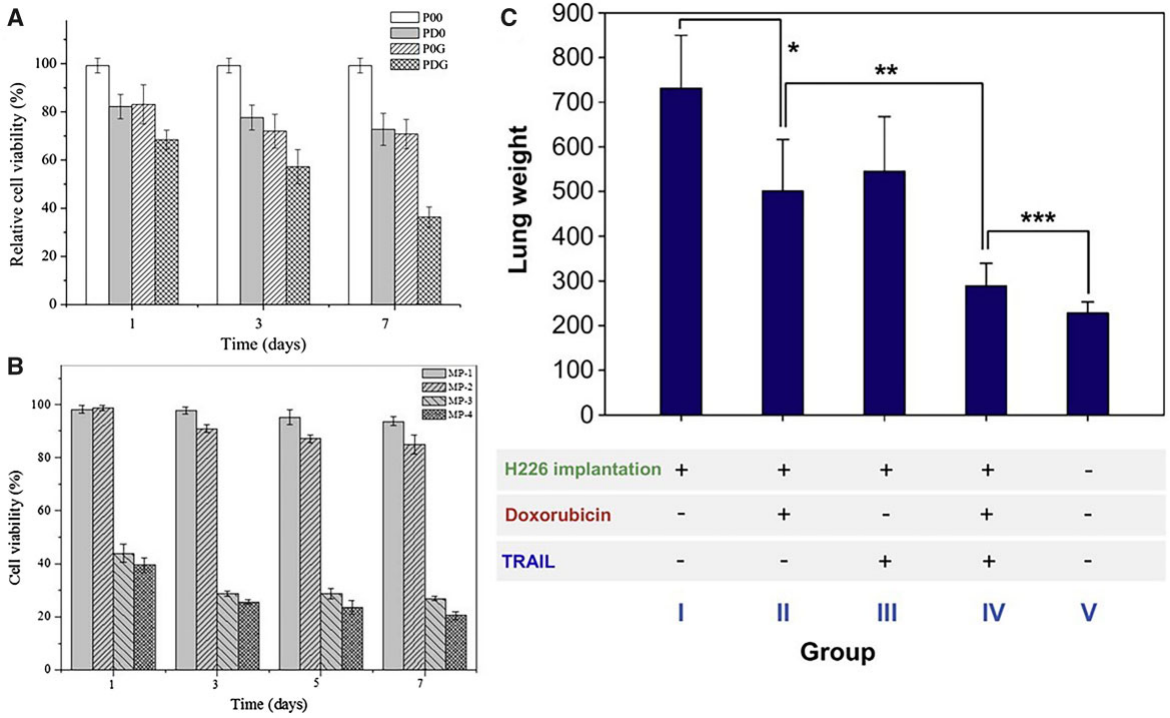
(**A** and **B**) The viability of the A549 cells after incubation in the supernatants from DOX-co-loaded MPs. (A): P00—control, PD0—DOX-loaded, P0G—p53-loaded, PDG DOX-co-p53-loaded PLGA MPs [90], (B): MP-1—control, MP-2—miR-519c-loaded, MP-3—DOX-loaded, MP-4—DOX-co-miR-519c-loaded PLGA MPs [107], and the lung weights after *in vivo* test of BALB/c mice 4 weeks after H226 cancer cells implementation and DOX/TRAIL-loaded PLGA MPs pulmonary administration (*P < 0.015 over group I; **P < 0.005 over group II; and ***P < 0.05 over group IV) [91]. All the pictures adapted with permission.

The other approach is to combine a chemostatic drug with another healing agent. The simplest combination of two chemostatics (DOX and PXT) was studied by Feng *et al*. [106] and showed that the combination of both drugs in the molar ratio 2/1 DOX/PXT in PLGA MPs inhibits tumor growth slightly more efficiently than MPs with DOX or PXT alone of similar drug loading. On the other hand, the chemotherapeutic drug may be combined with for example gene therapy or drugs inhibiting the removal mechanisms of chemotherapeutics from cancer cells, or with special apoptosis-inducing ligands binding selectively to the cancer cells. These strategies were used by Shi *et al*. [90], Wu *et al*. [107] and Kim *et al*. [91], respectively. In all these cases, researchers obtained MPs (Fig. 6G) that could affect cancer cell viability *in vitro* or reduce tumor growth *in vivo* (Fig. 7). Chen *et al*. [108] proposed another mixture of therapeutic agents of metformin—originally an antidiabetic drug—and DHA—an Omega 3 polyunsaturated fatty acid—that would suppress tumor metastasis by inhibiting NF-κB signal pathway and STAT3 pathways, changing the tumor inflammatory microenvironment. The porous MPs had suitable aerodynamic diameters (3.39–3.59 µm). The drug loading was not high: 1.79 ± 0.04% and 2.97 ± 0.07% for metformin and DHA, respectively, and the drug release rate was rather slow compared to other studies mentioned here: 58.57% for metformin and 47.20% for DHA over 10 days. However, MPs were able to successfully inhibit cancerous 4T1 cell adhesion, lung vascular permeability and prevent metastasis to the lungs *in vivo* in mice while metformin and DHA synergy was used. This work suggests that reversing the lung premetastatic niche may be the strategy to protect patients from tumor malignancy.

Other drugs also exhibited interesting results. Xiong *et al*. [92] encapsulated artesunate—a semisynthetic derivative of artemisinin that is extracted from *Artemisia annua—*in PLGA MPs (Fig. 6H). Among other works, the authors obtained an impressively high drug loading of 86.85 ± 2.55%. Furthermore, more than 90% of the drug was released within 8 days of incubation in Gamble’s solution. *In vitro* tests with A549 cells showed that artesunate-loaded MPs can reduce cell viability and inhibit cell migration in a wound healing assay. Wang *et al*. [110] conducted a very similar study using disulfiram, a drug used in alcoholism therapy that is now being investigated as an antitumor agent. Although the drug loading was clearly lower (4.09 ± 0.11%), the decrease in A549 cell viability was also clear. However, probably due to the limited supply of disulfiram within MPs, the antitumor effect was slightly lower, with 10.0% viability after incubation in 5 days release supernatants and 13.3% viability after incubation in 7 days supernatants for MPs loaded with artesunate and disulfiram, respectively. However, the supernatants of artesunate-loaded MPs were incubated with the cells for 48 h, as in the case of disulfiram-loaded it was 24 h. At any rate, both approaches seem promising for future investigation *in vivo.*

Zhu *et al*. [109] investigated the encapsulation and antitumor properties of oridonin, a natural herbal anti-inflammatory and antitumor compound, in PLGA MPs. MPs showed accurate aerodynamic properties *in vitro* (FPF up to 30%) and *in vivo*. The interesting part of the study is the very fast release rate—∼74% in 1 h. Surprisingly, oridonin-loaded MPs were more effective in inhibiting tumor growth in the lungs of rats than pure oridonin, which suggests that the polymeric carrier was able to deliver the drug more efficiently because of its proper aerodynamic properties.

Although PLGA seems to dominate in the field of polymeric inhalable DDSs, other polymers are investigated too. Cheng *et al*. [111] presented poly(ester-thioether) MPs loaded with a combination of erlotinib, a frequently used antitumor drug, and α-tocopheryl succinate, a vitamin E family. The drug loading was up to several percent—similar to the most recently investigated PLGA MPs—and showed drug release kinetics highly dependent on the MPs porosity (pores increased the release rate). *In vitro* tests showed the antitumor potential of all formulations, as porous MPs exhibited the highest inhibitory effect on A549 cells. *In vivo* experiments also showed the efficacy of the solution, as it was able to reduce tumor growth in BALB/c nude mice. However, the formulations were administered intratumorally, raising the question of whether a similar effect could be obtained when administered by inhalation.

In addition, inhalation is believed to be an optimal route of drug delivery in the treatment of pulmonary hypertension. Sildenafil is the common drug encapsulated in MPs for this purpose. Sildenafil-loaded PLGA MPs were investigated by Beck-Broichsitter *et al*. [112, 113]. The spray-dried formulations showed different release kinetics that varied depending on the manufacturing parameters and showed *ex vivo* that the absorption of sildenafil in the lungs is related mainly to the release of MPs. Shahin *et al*. [114] used the same drug, material and similar technique to obtain MPs and showed that the composition of the formulation is crucial in terms of EE—the results ranged from around 3 to around 95% due to simple changes in manufacturing conditions. Furthermore, it was shown *in vivo* that this DDS would lead to much higher concentrations in the lungs than commercially available sildenafil for oral administration.

Tuberculosis, lung cancers and infections related to chronic lung diseases are the main problems for which most polymeric MPs-based DDSs are investigated. Among other biodegradable polymers, the most popular is PLGA. However, there are also other studies dealing with other diseases and materials that seem to be valuable to mention. Another polymeric DDS for inhalation was based on the copolymer of PLA and monomethoxy poly(ethylene glycol) (mPEG) for gene therapy prepared by Terry *et al*. [115]. PLA is a biopolymer that appears less and less frequently in the literature nowadays because it is displaced by PLGA, but may still be applicable. The study aimed to create a DNA–polyethylene imine conjugate and then encapsulate it within PLA-mPEG MPs using the double emulsification method. The MPs obtained were porous and showed an impressively high EE, which is a promising result for future gene therapies. All the mentioned publications have been summarized in Table 4.

**Table 4.** Applications, manufacturing method and properties of MPs based on synthetic polymers used in pulmonary drug delivery

Polymer	API	Manufacturing method	MPs properties	Aerodynamic properties	References
PLGA	Rifampicin (tuberculosis)	Emulsification	Methanol in the O-phase—decreased roundness, **Size:** 4.47 ± 2.51 µm **EE:** 99.62 ± 0.12% **LE:** 19.92 ± 0.02%	**FPF:** 40.99 ± 4.58% (DCM: methanol = 70:30)	Nii *et al*. (2018) [85]
PLGA	Cinaciguat (pulmonary hypertension)	Single emulsification	**Size:** 12.39–18.85 µm **Morphology:** porous **EE:** 68–86%	**MMAD:** 4.82–6.17 µm **FPF:** 19.8–36.0%	Ni *et al*. (2017) [84]
PLGA	BAY 41-2272 (pulmonary arterial hypertension)	Premix membrane emulsification/single emulsification	Porosity up to 43.2%, smaller diameters thanks to premix membrane: (5.5–6.6) µm, **EE:** 70–89%	**MMAD:** 2.6–4.4 µm **FPF:** 25–30%	Zhang *et al*. (2020) [83]
PLGA	Budesonide/coumarin (tuberculosis)	Premix membrane emulsification/single emulsification	Lipid surface-modified, **Size:** 2.6–3.5 µm, **EE:** 81–89%	**MMAD:** 2.8–3.8 µm	Li *et al*. (2019) [86]
PLGA	Budesonide (asthma)	Premix membrane emulsification/single emulsification	PEG surface-modified, **Size:** 3.46 ± 0.05 µm **EE:** 89.11 ± 3.87% (chosen formulation)	***D*_ae_:** 3.82 ± 0.06 µm (chosen formulation)	Li *et al*. (2021) [87]
PLGA	Rifampicin (tuberculosis)	Spray-drying	Leucine-modified for non-spherical shape, **Size:** 10.65 ± 5.95 µm **EE:** 88.89 ± 1.40% **LE:** 15.46 ± 0.01%	**FPF:** 43.4 ± 5.7%	Takeuchi *et al*. (2018) [88]
PLGA	IDR-1018 (tuberculosis)	Double-emulsification	*N*-acetyl cysteine surface-modified for better mucus penetration, **Size:** 6.24 ± 1.04 µm **EE:** 59.34 ± 3.79% **LE:** 12.93 ± 1.44%	**MMAD:** 3.79 ± 1.04 µm **FPF:** 52.87 ± 5.11 %	Sharma *et al*. (2020) [89]
PLGA	Moxifloxacin (tuberculosis)	Vortex-induced single emulsification	**Size:** 3.16 ± 0.38 µm **Morphology:** round (chosen formulation)	**MMAD:** 2.85 ± 1.04 µm **FPF:** 72.77 ± 1.73% **GSD:** 3.10 ± 1.23	Vishwa *et al*. (2021) [94]
PLGA	Gatifloxacin (tuberculosis)	Single emulsification	**Size:** 4.5 ± 0.8 µm for the formulation with the highest EE and LE (89.6 ± 1.2% and 8.0 ± 0.5%, respectively)	**FPF:** 15.9% of the formulation with the highest EE	Marcianes *et al*. (2020) [95]
PLGA	Levofloxacin (cystic fibrosis)	Double emulsification + premix membrane homogenization	**Size:** 5.0 ± 1.7 µm **Morphology:** internal slightly porous, agglomeration **EE:** 23.1% **LE:** 10.5 ± 1.4%	**MMAD:** 7.1 ± 0.2 µm **FPF:** 30.2 ± 2.3%	Gaspar *et al*. (2019) [98]
PEG-PLGA	Tobramycin (lung bacterial infections)	Double emulsification	**Size (hydrodynamic diameter):** 0.896 ± 0.172 µm **PdI:** 0.18 ± 0.10 **EE:** 3.05 ± 0.40% **LE:** 0.15 ± 0.2%	–	Ernst *et al*. (2018) [99]
PCL	Azithromycin (pneumonia)	Double emulsification	**Morphology:** hollow MPs **Size (median diameter):** 5.76 ± 0.26 µm **EE:** 61.51 ± 0.83%, **LE:** 23.07 ± 0.31% (for formulation with the highest EE and LE values)	***D*_ae_:** 3.63 ± 0.22 µm	Kasten *et al*. (2016) [97]
PLGA	Bacteriophages (bacterial lung infections)	Double emulsification	**Size:** 8.0 ± 4.5 µm, 2.6 × 10^6^ p.f.u. (plaque forming units)/mg MPs, endotoxin units (EU): 0.078 ± 0.003 EU/mg **Morphology:** highly porous	***D*_ae_:** 2–5 µm	Agarwal *et al*. (2018) [100]
PLGA	Curcumin (idiopathic pulmonary fibrosis)	Double emulsification	**Size:** 11.58 µm, **Morphology:** porous **Span:** 4.197 **EE:** 95.8% **LE:** 16.9%	**MMAD:** 3.12 µm **FPF:** 13.41%	Hu *et al*. (2018) [101]
PSA	Curcumin (COPD)	Single emulsification	**Size:** 1.43 µm **Morphology:** non-porous, **EE:** 42.8 ± 0.7% **LE:** 11.0 ± 0.2%	–	Kwiecień *et al*. (2021) [102]
PEG-PMA	Alkaloids from *Alstonia scholaris* (anti-inflammatory, cough relief)	Double emulsification	**Size:** 1.6–3.3 µm **EE:** 64.3–72.9% **LE:** 3.0–4.4% – All increasing with longer PEG-chains	–	Jiang *et al*. (2021) [103]
PCADK/PLGA	Doxorubicin (DOX) (lung cancer)	Double emulsification	**Size:** 15.57 ± 8.86 µm **Morphology:** Porous and round (less PCAKD) or irregular (more PCAKD) **EE:** 77.22 ± 4.32%, **LE:** 2.49 ± 0.14% (chosen formulation)	***D*_ae_:** 2.48 ± 0.18 µm (chosen formulation)	Li *et al*. (2020) [104]
PLGA	Doxorubicin (DOX) (lung cancer)	Double emulsification	**Size:** 5.21 ± 0.95 µm **Morphology:** internally porous **EE:** 60.95 ± 0.88% **LE:** 5.54 ± 0.08%	**MMAD:** 2.58 ± 0.47 µm	Feng *et al*. (2015) [105]
PLGA	Doxorubicin (DOX), paclitaxel (PXT) (lung cancer)	Double emulsification	**Size:** 11.47 ± 2.71 µm **Morphology:** porous **EE:** 62.42 ± 0.88% (DOX) and 80.97 ± 0.99% (PXT) **Total LE:** 6.02 ± 0.08% (chosen formulation)	**MMAD:** 3.51 ± 0.83 µm	Feng *et al*. (2014) [106]
PLGA	Doxorubicin (DOX), miR-519c (lung cancer)	Double emulsification	**Size:** 47.4 ± 19.2 µm **Morphology:** porous **Zeta potential:** –3.0 ± 1.6 mV **EE:** 79.63 ± 3.21% (DOX) and 29.04 ± 1.33% (miR) **LE:** 0.796 ± 0.032% (DOX) and 0.023 ± 0.001% (miR)	***D*_ae_:** 8.97 ± 1.49 µm	Wu *et al*. (2016) [107]
PLGA	Doxorubicin (DOX), TRAIL (metastatic lung cancer)	Double emulsification	**Size:** 11.5 ± 0.4 µm **Morphology:** porous **EE:** 86.5 ± 6.5% (DOX) and 91.8 ± 2.4% (TRAIL)	–	Kim *et al*. (2013) [91]
PLGA	Doxorubicin (DOX), p53 (lung cancer)	Double emulsification	**Size:** 22.9 ± 11.8 µm **Morphology:** porous **Zeta potential:** 5.9 ± 5.8 mV **EE:** 88.2 ± 1.7% (DOX) and 36.5 ± 7.5% (p53), **LE:** 0.71 ± 0.03% (DOX) and 0.036 ± 0.008% (p53)	–	Shi *et al*. (2014) [90]
PLGA	Artesunate (non-small cell lung cancer)	Double emulsification	**Size:** 26.39 ± 2.12 µm, **Morphology:** porous **EE:** 30.27 ± 0.62% **LE:** 86.85 ± 2.55%	***D*_ae_:** 5.28 ± 0.42 µm	Xiong *et al*. (2021) [92]
PLGA	Metformin (Met), docosahexaenoic acid (DHA) (anti-tumor lung metastasis)	Double emulsification	**Size:** 20.38 ± 1.02 µm **Morphology:** porous **EE:** 53.68 ± 1.93% (Met) and 89.20 ± 2.07% (DHA) **LE:** 1.79 ± 0.04% (Met) and 2.97 ± 0.07% (DHA)	***D*_ae_:** 3.59 ± 0.09 µm	Chen *et al*. (2021) [108]
PLGA	Oridonin (non-small cell lung cancer)	Double emulsification	**Size (D_50_):** 11.6 ± 2.3 µm **Morphology:** smooth spheres with small pores **EE:** 81.5 ± 1.0%, **LE:** 9.3 ± 0.1%	***D*_ae_:** 2.7 ± 0.3 µm	Zhu *et al*. (2017) [109]
PLGA	Disulfiram (lung cancer)	Single emulsification	**Size:** 47.83 ± 13.21 µm **Morphology:** porous **Zeta potential:** –14.9 ± 4.7 mV **EE:** 81.84 ± 2.35%, **LE:** 4.09 ± 0.11%	***D*_ae_:** 8.31 ± 1.33 µm	Wang *et al*. (2017) [110]
Poly(ester-thioether)	Erlotinib, α-tocopheryl succinate (non-small cell lung cancer)	Single emulsification	**Size:** 12.9 µm—non-porous; 13.6 µm—porous, **LE (non-porous):** 7.1% (erlotinib), 6.2% (α-tocopheryl) **LE (porous):** 6.3% (erlotinib), 5.0% (α-tocopheryl)	–	Cheng *et al*. (2020) [111]
PLGA	Sildenafil (pulmonary hypertension)	Spray-drying	**Size:** 3.7–7.9 µm **Morphology:** non-porous, spherical **LE:** 5.1–37.3% (depending on formulation)	–	Beck-Broichsitter *et al*. (2017) [112], (2016) [113]
PLGA	Sildenafil citrate (pulmonary hypertension)	Double emulsification/spray freeze-drying	**Size:** 8.27 ± 1.70 µm **EE:** 94.20 ± 0.06% (chosen formulation)	**MMAD:** 4.52 ± 0.37 µm **FPF:** 25.33 ± 3.32% (chosen formulation)	Shahin *et al*. (2021) [114]

MPs, microparticles; EE, encapsulation efficiency; LE, loading efficiency; MMAD, mass median aerodynamic diameter; *D*_ae_, aerodynamic diameter; GSD, geometric standard deviation; FPF, fine particle fraction.

PLGA plays an important role in the manufacturing of inhalable formulations among any possible biopolymers. It was shown in several publications that it can be used successfully to reach the lungs after inhalation and release a reasonable amount of different drugs there. However, the degradation rate of PLGA, although it is adjustable by synthesis conditions and the substrates ratio, remains relatively slow. Even if it does not affect release kinetics, it may still lead to excessive accumulation in the case of prolonged therapy. Clearance mechanisms should be able to remove the polymer from healthy lungs. However, for patients with severe lung obstruction from for example CF or COPD, it would be encouraged to investigate faster degrading polymers, as they may provide an equally successful therapeutic effect with a lower risk of accumulation.

## Lipid-based MPs

Lipid-based carriers are one of the most popular particulate DDSs. This type of carrier is based on a hydrophobic lipid core enriched with active compounds, surrounded by stabilizing surfactant molecules [4, 116]. These carriers are made of lipids that are solid at room and body temperature (usually with a melting point above 45°C), which not only enhances the stability of drug molecules but also allows administration with DPI [117]. The lipids used to manufacture carriers are biodegradable and occur naturally in the human body; therefore, lipid-based DDSs are considered non-toxic and safe for use [4, 117]. The vast diversity of available lipids (e.g. fatty acids, fatty acid alcohols, triglycerides, steroids, esters or waxes) enables the precise selection of the optimal lipid for a specific purpose in terms of physicochemical properties, efficient drug loading of both hydrophilic or hydrophobic compounds or biological performance [4, 117, 118]. Their efficient degradation *in vivo* into natural, non-toxic products that can be metabolized by cells and high loading capacity are their most pronounced advantages over commonly used polymeric particles [116].

Although colloidal solid lipid nanoparticles (SLNs) and nanostructured lipid carriers (NLCs) are the most popular lipid-based DDS, they are not suitable for inhalation in a form of DPI. Therefore, solid lipid MPs and lipid NP agglomerates are being developed for the direct delivery of active compounds to the lungs [117, 119, 120].

In general, solid lipid microparticles (SLMs) have a spherical shape with diameters in the range of 1–1000 µm [121]. However, studies on the aerodynamic properties of SLMs and their deposition in the lungs showed that for most SLMs, the optimal size range for successful pulmonary delivery is similar to other formulations, i.e. between 1 and 5 µm [122].

Numerous approved drugs have been developed for the treatment of both systemic (i.e. diabetes) and pulmonary (i.e. asthma, COPD, infections and cancer) diseases [122]. A wide variety of APIs, their doses, lipid excipients and manufacturing methods have been described in detail providing a solid knowledge base on the subject [14].

Similarly, to polymeric MPs, SLM can be manufactured using various emulsification-based methods in which the organic phase consists of lipid loaded with API while an aqueous solution of ionic or non-ionic surfactants constitutes the external water phase [116, 121]. Because lipids used for SLM fabrication have lower melting temperatures than those of natural or synthetic polymers, the use of the hot emulsification technique is possible. In this method, both organic and water phases are heated to temperatures exceeding the melting point of the lipid by 10–20°C and API is dispersed in the melted lipid. The organic phase is then emulsified into the external water phase using high sheer mixing and cooled to solidify the SLM obtained SLM [123–125]. The hot oil-in-water emulsification technique is appropriate for thermally stable APIs, and its main advantage over other emulsification modes is that the use of potentially toxic organic solvents is avoided.

**Figure 8.**
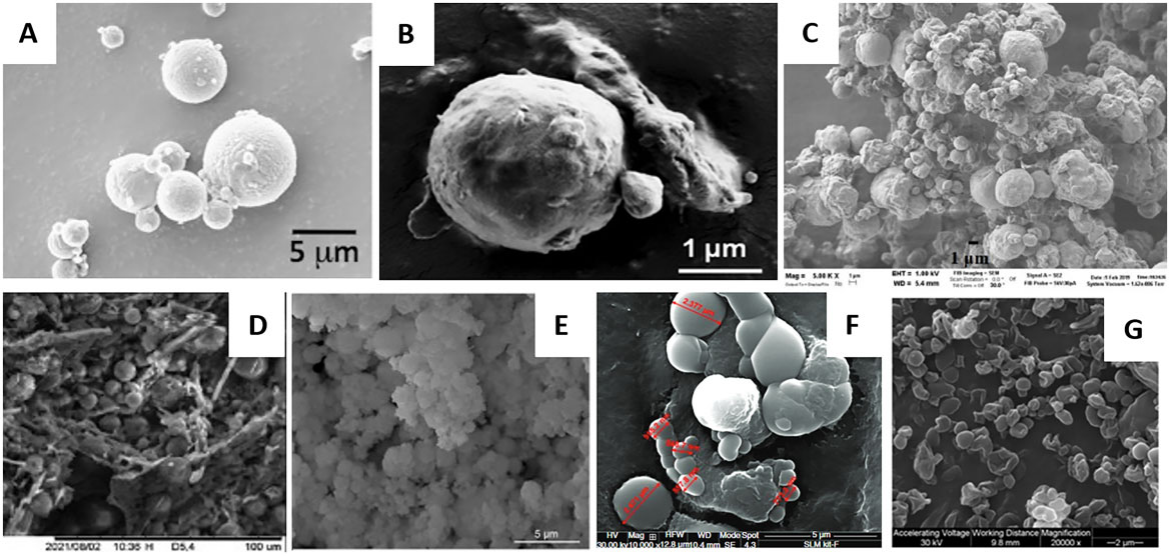
SEM images of solid lipid MPs: (**A**) dilyceryl behenate MPs loaded with budesonide [123]; (**B**) glycerol behenate MPs loaded with budesonide [124]; (**C**) glyceryl dibehenate MPs loaded with solubutomal sulfate [126]; (**D**) glyceryl behenate MPs loaded with quercetin [127]; (**E**) tristearin with PEG modification loaded with cisplatin [128]; (**F**) glycerol tripalmitate with chitosan modification loaded with fluticasone propionate [129]; (**G**) phospholipids loaded with amphotericin B [130]. All the pictures adapted with permission.

Mezzena *et al*. [123] used the hot oil-in-water emulsification method for the fabrication of SLM loaded with budesonide. Diglyceryl behenate was used as a matrix material, while Pluronic F-68 served as a surfactant. Emulsification was carried out at 90°C using high sheer mixing at 10 000 rpm for 2 min followed by rapid cooling by submersion in an ice bath under continuous magnetic stirring. The dry powder was obtained after the spray-drying process. The SLMs obtained were irregular in shape with a median diameter of 3.45 ± 0.27 µm (Fig. 8A). Encapsulation within SLM decreased the *in vitro* release kinetics of budesonide and allowed sustained release of API for up to 6 h in phosphate buffered saline and simulated lung fluid. Additionally, SLMs were significantly less likely to be retained within the DPI apparatus compared to free budesonide, which was attributed to reduced particle roughness and adhesion. The FPF of SLM was 21.1 ± 0.6%, which at that time corresponded to other DPI-based formulations (FPF below 20%). The developed formulation was later improved by Upadhyay *et al*. [124] and the fabricated SLMs were characterized by FPF >30%. However, since both formulations were similar in composition and properties (Fig. 8B), the enhanced aerosolization performance of the latter SLM could also be attributed to the use of a newer and more effective DPI device.

Recent studies on the use of the hot oil-in-water emulsification method for SLM fabrication were followed by the spray-drying process to obtain the DPI formulation as presented by Ignjatović *et al*. [126]. Glyceryl dibehenate and stearyl alcohol mixed with SAS as API were used as the organic phase, while Poloxamer 188 served as a surfactant. Both the lipids and the water phases were heated to 70–90°C. The water phase was slowly poured into the organic phase (to minimize lipid loss during material transfer) and mixed at more than 13 000 rpm for at least 2 min. In contrast to the previously described SLM, the obtained emulsion was allowed to cool down to room temperature slowly just under magnetic stirring (no rapid cooling in ice was applied). Drug loading was higher in the case of glyceryl dibehenate SLM (maximum of 13.99 ± 0.57%); however, the SLM were irregular or spherical in shape and rather porous, which was believed to be advantageous in terms of pulmonary delivery (Fig. 8C). The average particle size was between 3.94 and 7.09 µm, FPF ranged from 19.93 ± 1.18% to 38.04 ± 4.84%, and sustained release of SAS was provided for up to 2 h. The research presented has great value since several manufacturing parameters (i.e. lipid type, surfactant concentration, mixing speed and duration, washing and spray-drying conditions) were tested, and their influence on the properties of SLM was evaluated in detail.

DPI formulations based on SLM can also be obtained by freeze-drying purified emulsions. Rosita *et al*. [127] recently fabricated SLM loaded with antioxidant and anti-inflammatory quercetin using hot oil-in-water emulsification followed by particle freeze-drying. The developed method resulted in a high yield of SLM (above 88%). The particles were spherical (Fig. 8D) with particle sizes in the range of 1.79 ± 0.13 µm to 1.91 ± 0.11 µm (as determined by dynamic light scattering). They also showed high encapsulation efficacy (58.41 ± 4.10% to 88.48 ± 4.20%, resulting in drug loading between 8.57 ± 0.77% and 10.94 ± 0.50%), and good aerosolization properties. Reczyńska *et al*. [125] developed fatty acid-based MPs loaded with the anticancer drug—PXT and superparamagnetic iron oxide nanoparticles (SPION) for the treatment of lung cancer. The MPs were obtained via hot oil-in-water emulsification followed by immediate cooling of the emulsion in liquid nitrogen and freeze-drying of the purified MPs. The MPs were spherical and porous, with high PXT loading efficacy, and mobile in the magnetic field due to SPION incorporation. Their anticancer efficacy was confirmed *in vitro* in contact with malignant lung epithelial cells (A549).

Another commonly used method used in the fabrication of SLM is high-pressure homogenization (HPH). This technique can be applied for the large-scale production of lipid-based DPIs, although it is predominantly designed to mix, stabilize, and reduction of the droplet size of various emulsions. In general, liquid (called a premix, usually a coarse emulsion or dispersion) under high pressure (50–500 MPa) passes through a thin nozzle. During this passage, larger droplets break down into smaller, usually uniform particles [131].

Levet *et al*. [128] developed cisplatin-loaded lipid MPs based on tristearin for lung cancer treatment. HPH was employed to reduce the size of cisplatin that was later mixed with tristearin dissolved in isopropanol, and the obtained suspension was spray-dried into a DPI formulation. To avoid rapid mucociliary clearance, the MPs were modified with PEG to provide them with a stealth coating. Depending on a formulation (i.e. the composition of MPs), the developed MPs were spherical or more irregular in shape (Fig. 8E) with MMAD ranging from 2.0 ± 0.2 µm to 2.5 ± 0.3 µm (44–80% of particles with diameters below 5 µm) and FPF between 24.2 ± 6.3% and 50.3 ± 5.8%. Encapsulation of cisplatin within lipid/PEG MPs significantly slowed down drug release and prolonged it for more than 24 h (compared to burst dissolution of almost 100% cisplatin within the first hour in the case of free drug). Selected formulations were later tested *in vivo* using a mouse model. DPIs were administered directly to the mice’s tracheas, while intravenous injection and endotracheal nebulization of cisplatin solution were used for comparison. It was found that in all inhaled experiments, the concentration of cisplatin in the lung was significantly higher than in the case of intravenous injection. Modification of the surface with PEG significantly increased MP lung retention that was sustained for up to 7 h [132].

The surface modifications of MPs are not only aimed at evasion of recognition by the immune system and clearance mechanisms, but can also provide a better mucoadhesion to prolong MP activity. Amore *et al*. [129] prepared glycerol tripalmitate MPs loaded with fluticasone propionate (FP, used in COPD treatment) using the ethanolic precipitation technique. In this method, soybean lecithin (surfactant) dissolved in ethanol was added to FP mixed with melted lipid. The resulting solution was further dispersed in hot water containing the chitosan derivative under vigorous stirring followed by high pressure homogenization and rapid cooling in an ice bath. The MPs obtained were rather spherical (Fig. 8F) with diameters of 1.5–2.5 µm and a strongly positive surface zeta potential (more than 20 mV in water or 0.9% NaCl). Chitosan coating was believed to increase mucoadhesiveness of MPs; however, only 12.4% by weight of MPs adhered to a cellulose membrane soaked with mucin (simulating the pulmonary epithelial surface) after washing. The FP loading was equal to 7.47% and the drug release tests showed that more than 40% of the cargo was released from the MPs within the first 6 h of incubation. On the other hand, the further release was decreased and after 48 h of incubation only around 50% of FP was still entrapped within the MPs. MPs were not cytotoxic to bronchial epithelial cells (16HBE).

Gomez *et al*. [132] recently presented another approach for the fabrication of lipid-based MPs for patients with CF [130]. The study showed that it was possible to obtain inhalable DPI by spray drying a novel proliposome loaded with antifungal amphotericin B. Synthetic phospholipids were dissolved with amphotericin B in methanol and co-spray dried at different conditions. The resulting powders were spherical and uniform with smooth surfaces (Fig. 8G) and their diameters ranged from 1.1 to 1.3 µm (no particles > 5 µm). The drug load ranged from 0.146 to 0.155 mg of amphotericin B per 1 mg of MPs. The co-spray-dried powders had sufficient aerosolization properties (FPF between 74.5% and 82.2%) and were cytocompatible with H358 and A549 lung epithelial cells at a concentration below 100 µM (Table 5).

**Table 5.** Applications, manufacturing method and properties of solid lipid MPs

Lipid	API	Manufacturing method	MPs properties	Aerodynamic properties	References
Dilyceryl behenate	Budesonide (asthma, COPD)	Hot emulsification/spray drying	**Morphology:** irregular, slightly spherical **Size (DLS):** 3.45 ± 0.27 µm	**FPF:** 21.1 ± 0.6%	Mezzena *et al*. (2009) [123]
Glycerol behenate	Budesonide (asthma, COPD)	Hot emulsification/freeze-drying	**Morphology:** rather spherical, some irregularities visible on the surface **Size (SIOS):** 1.6–1.8 µm (mean)	**FPF:** 30.00 ± 1.15%	Upadhyay *et al*. (2012) [124]
Glyceryl dibehenate (GB) or stearyl alcohol (SA)	Salbutamol sulfate (asthma, COPD)	Hot emulsification/spray drying	**Morphology:** spherical, rather uniform MPs **Size (LLS):** 3.94–7.09 µm **LE:** 0.87–13.99% (depending on lipid type and washing procedure)	**FPF:** 19–38% **MMAD:** 2.94–3.56 µm (depending on lipid type and washing procedure)	Ignjatović *et al*. (2021) [126]
Lauric acid (LAU)	Paclitaxel and superparamagnetic iron oxide nanoparticles (SPION) (lung cancer)	Hot oil-in-water emulsification	**Morphology:** spherical and porous **Size:** 1.9–3.6 μm **Zeta potential:** –9.9±0.7 to –12.5±0.7 mV **EE:** 99.6±0.3% **LE:** 4.9±0.1%)	–	Reczyńska *et al*. (2020) [125]
Glyceryl behenate	Quercetin (pneumonia)	Melt emulsification/freeze-drying	**Morphology:** rather spherical and smooth **Size (measurements based on optical microscopy images):** 1.79 ± 0.13–1.91 ± 0.11 µm **LE:** 8.57 ± 0.77–10.94 ± 0.50%	–	Rosita *et al*. (2022) [127]
Tristearin + PEG modification	Cisplatin (cancer)	High pressure homogenization/spray drying	**Morphology:** irregular **Size (measurements based on SEM images):** 1.6–4.2 µm (median)	**FPF:** 24.2–50.3% (bulk cisplatin—4.2%) **MMAD:** 2–2.4 µm	Levet *et al*. (2016) [128]
Glycerol tripalmitate + chitosan modification	Fluticasone propionate (COPD)	Ethanolic precipitation/high pressure homogenization/freeze-drying	**Morphology:** slightly irregular, rather spherical **Size (DLS):** 1.5–2.5 µm **LE:** 7.47%	–	Amore *et al*. (2017) [129]
Phospholipids (DPPC, DPPG)	Amphotericin B (fungal infections, CF)	Spray drying of proliposomes	**Morphology:** rather spherical, slightly irregular **Size (measurements based on SEM images):** 1.10–1.36 μm **LE:** 1.2–1.67%	**FPF:** 74.5–82.2%	Gomez *et al*. (2020) [130]

DLS, dynamic light scattering; SIOS, scanning ion occlusion sensing; LLS, laser light scattering; DPPC, dipalmityolphosphatidylocholine; DPPG, dipalmitoylphosphatidylglycerol; MPs, microparticles; EE, encapsulation efficiency; LE, loading efficiency; MMAD, mass median aerodynamic diameter; FPF, fine particle fraction.

## Novel approaches

MPs delivered to the lungs are at risk of removal through natural cleansing processes such as mucociliary cleansing or phagocytosis. To solve this problem, NPs are encapsulated in the MPs (also called Trojan or NP agglomerates). Due to the appropriate aerodynamic diameter, MPs can be deposited in the lower respiratory tract. Then MPs can degrade and the NPs released can penetrate mucus and avoid macrophages [133–135].

### Lipid NP assemblies

Although techniques for the fabrication of solid lipid MPs have already been well established and numerous formulations have been developed, several limitations (i.e. possible low drug loading capacity) force the search for new solutions. SLN or NLC, being another dynamically developing group of lipid-based drug carriers, cannot be used for direct pulmonary delivery as DPI; however, those NPs can be formulated into MP-like agglomerates or assemblies. Several of such formulations have already been reported in the literature.

Maretti *et al*. [136] developed DPI based on SLN assemblies (SLNas) for direct intramacrophagic antitubercular therapy. SLNs were obtained using the hot emulsification/ultrasonic homogenization method based on stearic acid and rifampicin using sodium taurocholate as a surfactant. The resulting NPs were purified and freeze-dried under different conditions to obtain SLNas. The SLNas were irregular in shape, as evidenced by transmission electron microscopy, with particle size within 0.6–1.7 µm, high polydispersity index (PdI > 0.33) and strongly negative surface zeta potential (approximately-45 mV). However, they were characterized by efficient drug encapsulation (> 45%) and high rifampicin loading (11.8–15.9%). The aerodynamic properties of DPI *in vitro* were strongly improved by increasing the sample dilution during freezing of the SLN suspension, rapid freezing and avoidance of the use of cryoprotectants (the highest respirable fraction was ∼60%). The efficacy of macrophage targeting was further increased by surface functionalization of previously described SLNas with mannose derivatives, as mannose receptors are overexpressed by infected alveolar macrophages. Approximately 80% of surface-modified SLNas were incorporated by macrophages, while in the case of unmodified SLNa, it was only 40% and 20% for free rifampicin [137]. *In vivo* studies in mice models evidenced a significantly higher retention of mannose-modified SLNa in the lungs, which was attributed to the more efficient phagocytosis of drug carriers by alveolar macrophages [138].

Nemati *et al*. [139] also used the hot emulsification method for the production of glyceryl behenate solid lipid NPs loaded with ethambutol hydrochloride (anti-tuberculosis drug). SLNs were rather uniform (PdI in the range of 0.243–0.502, depending on a formulation) with average hydrodynamic diameters below 100 nm and high drug loading (14.7–29.7%). For optimal delivery to the lungs, SLNs were spray dried in the presence or absence of an excipient (namely, mannitol) to obtain the DPI formulation. As prepared, the MPs were spherical with a mean size distribution of 1–2 µm. The presence of mannitol significantly increased the bulk volume of the MPs (1.95 ± 0.07 cm^3^ for MPs without mannitol and 2.50 ± 0.00 cm^3^ for MPs containing mannitol); however, the presence of an excipient improved the aerosolization performance of MPs (FPF of 23.98 ± 0.38% for MPs and 30.91 ± 0.77% for MPs containing mannitol).

Another approach was adapted by Amore *et al*. [140] who fabricated solid lipid NPs loaded with salmeterol xinafoate (SX, bronchodilator used in COPD treatment) which were then coated with sodium alginate to obtain mucoadhesive lipid MPs. Similar to previously described research, glyceryl distearate-based SLNs were produced using a hot emulsification method utilizing ultrasonic homogenization for proper dispersion of the lipid phase. The purified SLNs were then introduced to an aqueous solution of sodium alginate under mechanical stirring followed by crosslinking of alginate in calcium chloride solution. As prepared, the MPs were freeze-dried to obtain DPI formulation. The average size of the SLN was 133 nm (unloaded SLN) or 278 nm (SX-loaded SLN), and both types of NPs were characterized by a strongly positive zeta potential (more than 33 mV). Encapsulation of SLN in sodium alginate resulted in the formation of MPs with diameters of around 3.3 µm and negative zeta potential (below –20 mV). The MPs exhibited excellent aerosolization properties with FPF of 75 ± 7%, MMAD of 3.65 ± 1.29 µm, and were cytocompatible with bronchial epithelial cells (16HBE) (Table 6).

**Table 6.** Applications, manufacturing method and properties of solid lipid nanoparticles assemblies

Lipid	API	Manufacturing method	MPs properties	Aerodynamic properties	References
Stearic acid	Rifampicin (tuberculosis)	Hot emulsification/freeze-drying	**Morphology:** irregular **Size (DLS):** 0.6–1.7 µm **LE:** 11.8–15.9%	–	Maretti *et al*. (2016) [136]
Glyceryl behenate	Ethambutol hydrochloride (tuberculosis)	Hot emulsification/spray drying	**Morphology:** spherical, rather smooth **Size (measurement based on SEM images):** 1–2 µm	**MMAD:** 4.15–5.63 µm **FPF:** 23.98 ± 0.38–30.91 ± 0.77%	Nemati *et al*. (2019) [139]
Glyceryl distearate	Salmeterol Xinafoate (COPD)	Hot emulsification/coating with sodium alginate	**Size (DLS):** 3.3 µm	**MMAD:** 3.65 ± 1.29 µm **FPF:** 75 ± 7%	Amore *et al*. (2019) [140]

MPs, microparticles; LE, loading efficiency; MMAD, mass median aerodynamic diameter; FPF, fine particle fraction; DLS, dynamic light scattering.

### Composite MPs and NPs agglomerates

Many challenges of drug delivery directly to the lungs (e.g. appropriate size for inhalation being at the same time the most easily phagocytosed by macrophages) lead to new ideas in the field. An example of the newest approach is creating composite MPs made of at least two different materials to take advantage of the properties of both.

**Figure 9.**
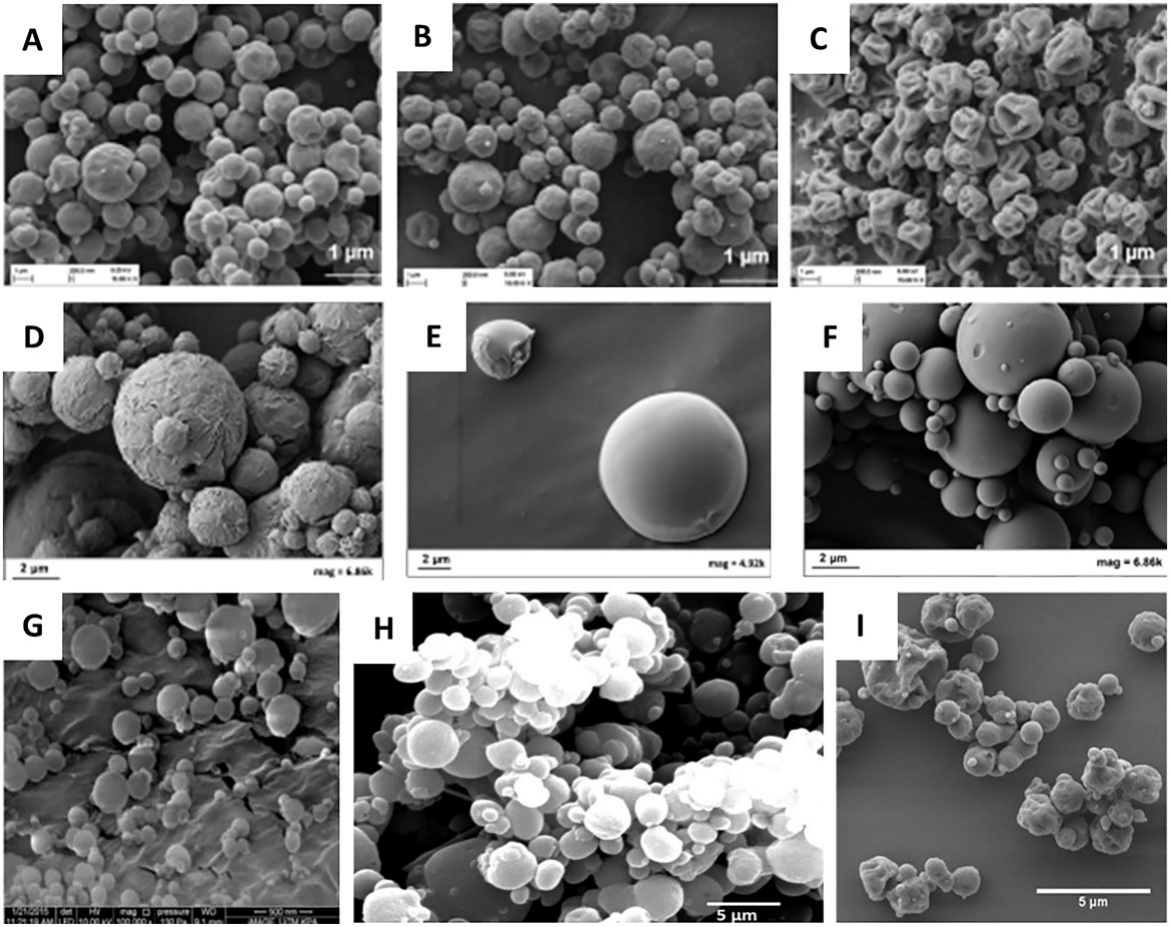
SEM images of inhalable composite NPs embedded within MPs: (**A**) Azi/NAC, (**B**) Cipro/NAC, (**C**) Tobr/NAC [143]; (**D**) lactose monohydrate based Trojan NPs, (**E**) trehalose-based Trojan NPs, (**F**) raffinose-based Trojan NPs [144]; (**G**) chitosan NPs/lactose-PEG3000 [145]; (H) PLGA/PLGA [141]; and (I) PLGA/mannitol [142]. All the pictures adapted with permission.

In some cases, the approach invented for SLNas aims to be transferred to other materials. For example, Chishti and Dehghan [141] used the same strategy to obtain PLGA NPs of around 200 nm diameters by single emulsification assisted by sonification. Then they created their assemblies to the right size for alveolar accumulation by spray drying (Fig. 9H). This formulation was found to be capable of decay after inhalation to slightly larger NPs (from 214.33 ± 4.01 µm to 223 ± 4.31 µm for fresh and redispersed NPs, respectively) and with significantly higher PdI (an increase from 0.123 ± 0.014 to 0.432 ± 0.031). As NPs were able to release docetaxel for 144 days and penetrate mucus and accumulate in cells, it seems that this technique could be promising for an inhalable formulation of any material. Baghdan *et al*. [142], on the other hand, decided to manufacture PLGA NPs and agglomerate them by spray drying with mannitol (Fig. 9I), which can quickly dissolve in lung fluids, rapidly redispersing the NPs. In the formulation, they encapsulated curcumin with very high EE (around 95%) for phototherapy of lung cancer. It was shown that irradiating A549 cells with 457 nm wavelength light increases their toxicity against cancer cells. If the result can be obtained *in vivo* and the redispersed PLGA NPs can efficiently penetrate the mucus to accumulate in cancer cells, it could be the new direction of lung cancer therapy. In another study, Lababidi *et al*. [143] manufactured an inhalable MP formulation using spray drying that combined multiple drugs: antibiotic (Tobr (Fig. 9C), Cip (Fig. 9B) or Azi (Fig. 9A)), *N*-acetylcysteine (NAC) and curcumin. The curcumin was encapsulated in PLGA NPs which were manufactured using a microfluidic system. The best aerodynamic properties had Tobr/NAC/NPs (FPF: 87.21 ± 8.63%). Moreover, the encapsulation of Tobr improved its antibacterial effect. Data show that co-formulation of Tobr with NAC loaded with NPs induced 80% bacterial killing at ∼1 µg/ml, while for the same concentration of the free drug, only 20% killing could be observed. Umerska *et al*. [144] encapsulated lipid nanocapsules-based Trojan particles in carbohydrate-based MPs. Lactose (Fig. 9D), trehalose (Fig. 9E) and raffinose-based Trojan particles (Fig. 9F) were easily dispersed as aerosols with MMAD between 5.3 ± 0.1 μm and 6.2 ± 0.1 μm using a DPI. The smallest MMAD for MPs was obtained by Alhajj *et al*. [145] for chitosan NPs in lactose-PEG3000 MPs (Fig. 9) (4.25 ± 0.85 μm for chosen formulation) (Table 7).

**Table 7.** Applications, manufacturing method and properties of composite NPs within MPs

Materials	API	Manufacturing method	MPs properties	Aerodynamic properties	References
PLGA NPs/l-leucine	Nanoparticles loaded with curcumin (NPs)/*N*-acetylcysteine (NAC)/azithromycin (Azi), tobramycin (Tobra) or ciprofloxacin (Cipro) (cystic fibrosis)	Spray drying	**Morphology:** spherical shape	**MMAD:** 2.56 ± 0.04 µm (*Cipro/NAC/NPs*); 2.43 ± 0.15 µm (*Tobra/NAC/NPs*); 2.51 ± 0.06 µm (*Azi/NAC/NPs*) **FPF:** 64.48 ± 3.22% (*Cipro/NAC/NPs*); 87.21 ± 8.63% (*Tobra/NAC/NPs*); 68.83 ± 6.11% (*Azi/NAC/NPs*) **GSD:** 1.62 ± 0.02 (*Cipro/NAC/NPs*); 1.47 ± 0.24 (*Tobra/NAC/NPs*); 1.58 ± 0.04 (*Azi/NAC/NPs*)	Lababidi *et al*. (2020) [143]
Lipid NPs (polyoxyl 15 hydroxystearate, hydrogenated lecithin and caprylic/capric acid triglycerides)/carbohydrate (lactose monohydrate, trehalose dihydrate or raffinose pentahydrate)	–	Spray drying	**Morphology (*lactose monohydrate-based Trojan NPs*):** spherical in shape with rough surfaces, hollow, had holes and cracks, the shell was composed of plate-like elements **Size (*trehalose based Trojan NPs*):** 6.11±0.03 –8.34±0.00 μm **Morphology (*raffinose-based Trojan NPs*):** spherical with dimpled surfaces	***Lactose monohydrate based Trojan NPs*** **MMAD:** 5.4±0.1 μm **FPF:** 29.0 ±1.0% **GSD:** 2.3 ± 0.2 (lactose monohydrate-based Trojan NPs) ***Trehalose-based Trojan NPs:*** **MMAD:** 6.1 ± 0.3 μm **FPF:** 16.7 ± 2.8% **GSD:** 3.0 ± 0.1 ***Raffinose-based Trojan NPs:*** **MMAD:** 5.3 ± 0.1 μm **FPF:** 23.4 ± 1.2% **GSD:** 2.4 ± 0.2	Umerska *et al*. (2020) [144]
Chitosan NPs/lactose-PEG3000	–	Spray drying	**Size:** 5.43 ± 0.10 μm **Morphology:** non-spherical shape with a circularity	**MMAD:** 4.25 ± 0.85 μm **FPF:** 36.96±4.64% **GSD:** 5.14 ± 0.35 **ED:** 10.11 ± 1.69 mg (for chosen formulation)	Alhajj *et al*. (2020) [145]
PLGA	Docetaxel	Sonification-assisted single emulsification for NPs and spray-drying for MPs, respectively	**Size:** 3.74 ± 0.06 µm ***The size of NPs:*** *214.33 ± 4.01 nm,* ***PdI:*** *0.123 ± 0.014,* ***EE:*** *58.2%,* ***Zeta potential:*** *–34.8 mV*	**MMAD:** 3.74 ± 0.11 µm **FPF:** 42.96 ± 1.66%, **ED :** 92.03 ± 0.12%, **GSD:** 1.87 ± 0.05	Chishti and Dehghan (2020) [141]
PLGA/mannitol	Curcumin	Emulsification with nanoprecipitation for NPs and spray-drying for MPs	**Size:** 0.5–4 µm ***Size for NPs:*** *181.20 ± 11.52 nm* ***PdI:*** *0.08 ± 0.02* ***Zeta potential:*** *–4.63 ± 0.13 mV* ***EE:*** *94.38 ± 0.64%*	**MMAD:** 3.02 ± 0.07 μm **FPF:** 64.94 ± 3.47%, **GSD:** 1.74 ± 0.16	Baghdan *et al*. (2019) [142]

MPs, microparticles; NPs, nanoparticles; EE, encapsulation efficiency; MMAD, mass median aerodynamic diameter; ED, emitted dose; GSD, geometric standard deviation; FPF, fine particle fraction; PdI, polydispersity index.

## Challenges and future perspectives

Pulmonary drug delivery is an alternative route of drug administration. However, all dry powders, before being approved must pass numerous tests. The process of obtaining the right DPI system is long and complicated. First, the choice of materials plays a crucial role. Different formulations may be based on previously used or novel polysaccharides, solid lipids or synthetic and natural polymers, as well as various combinations of them. The choice should mostly concern the type of drug to be encapsulated and the area of delivery—there is a significant difference between rapid and sustained release or whether the particles should or should not be absorbed by macrophages.

The next challenge is to manufacture the MPs of the right characteristic from both physicochemical and aerodynamic points of view. Among the possible MP manufacturing methods, different variants of emulsification and spray drying are, without a doubt, the most common. Although microfluidics is included in these techniques in several reviews [79–81], the method does not appear very often in recent studies related to inhalable MPs. Despite the huge advantages of microfluidics over other approaches, such as a very narrow size distribution and high repeatability of generated MPs, the main problem is size. There are commercially available systems dedicated to the manufacture of PLGA MPs; however, the smallest droplet that can be obtained generates MPs too large to be inhaled [146, 147]. The generation of smaller MPs might be possible by manipulating parameters such as the polymer concentration, but the question is if it will be possible to reduce the size enough. The possibility of using such devices may greatly improve the results. In particular, the regular emulsification method is hardly scalable to industrial manufacturing. This creates a niche for microfluidics producers to adjust their products to create smaller MPs. The task is not easy, as it requires reducing the size of the nozzle to extremely thin.

**Figure 10.**
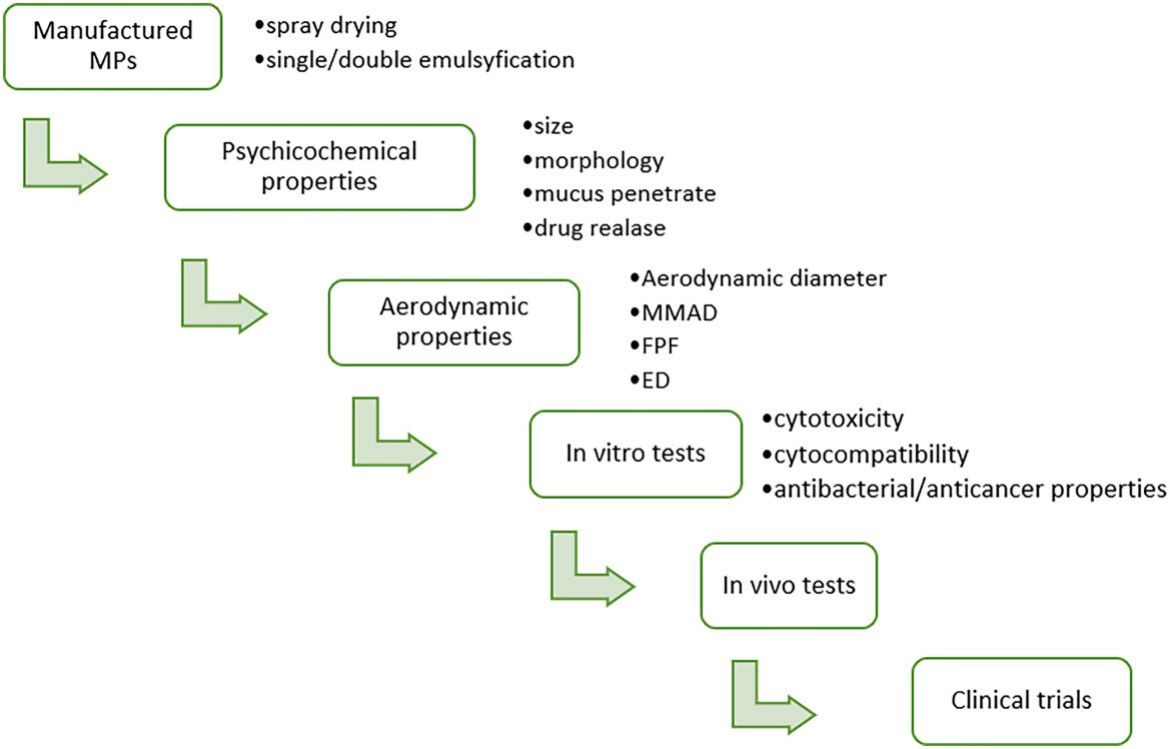
Multistage process of formulation inhalable MPs.

Designing an inhalable formulation is a multistage process, and each of the stages requires its own specifications (Fig. 10), which may sometimes be difficult to obtain simultaneously. In the following sub-chapters, the most common problems faced in the field and the research trends to overcome them have been described accordingly to these stages.

### Physicochemical properties

In addition to those physicochemical properties mentioned above (Section ‘Requirements for microparticles as dry powders for inhalation’) such as density, surface change, change or size of MPs, they must penetrate the mucus. Several physical and chemical factors affect mucoadhesion between the mucous-like surface and the polymer, such as molecular weight, plasticity, cross-linking, swelling, spatial conformation, concentration, surface charge, hydrogen bonding sites in the polymer and pH of the mucoadhesion interface [148]. One of the biggest challenges is to determine whether MPs can pass through the mucus. However, mucus investigation is not easy to carry out. Interactions with therapeutic formulations are not only related to the requirements described in Section ‘Requirements for microparticles as dry powders for inhalation’, but there may also be some changes in the lung environment caused by obstructive diseases or infections that could change the behavior of the formulation. Furthermore, it is difficult to possess a reasonable amount of native mucus for research purposes. Obtaining it from the human lungs involves invasive sampling procedures or bronchoscopy that require qualified personnel and/or ethical permits. One of the possible solutions to avoid this problem is the use of alternative sources of mucus, e.g. cell- or animal-derived mucus or synthetic mucus. These approaches allow to obtain a large amount of mucus. However, they are not free from limitations. Animal-derived mucus (e.g. from pigs), e.g. is different from that of humans and lacks a systemic comparison with it. Cell-derived mucus could be produced directly in the laboratory, where the inhalable formulation is investigated, but is contaminated with cells at the same time and it is difficult to distinguish the layers of mucus and cells. Synthetic formulations are commercially available, but do not perfectly mimic properties such as rheology. It seems that the most suitable model for research is the last one anyway. In this method, mucins—the main component of mucus—are harvested from porcine gastric and intestinal mucus and used to obtain hydrogels with rheology and barrier properties similar to the natural composition. As native mucus is highly heterogeneous and its properties are affected by many aspects, the use of purified commercially available mucins seems to be a reasonable approach to test DDSs with results comparable with other studies around the world. To track the fate of MPs in such a synthetic mucus, video microscopy is used. On the other hand, industrial purification causes some irreversible loss of the ability to form cross-linked gels, so the mimicking of native mucus is limited [149].

### Aerodynamic properties

As mentioned above, the physical diameter of the MPs can sometimes be much different from the aerodynamic one. Therefore, the physical size evaluation is not sufficient in terms of inhalation delivery. However, a significant number of recent studies have not taken into account the aerodynamic properties of their formulations. This may lead to bias in the results, as MPs that appear to be perfect for inhalation may, in fact, be unable to deposit in the lungs after inhalation. In light of this, it should be recommended to use multistage cascade impactors (CIs) in any new formulation dedicated this purpose. CIs are devices dedicated to that problem and are defined for the measurement of aerosol aerodynamic particle size distribution. Such an equipment works on the principle of inertial size fractionation, so the aerodynamic diameter is evaluated directly. Moreover, the construction allows quantification of the mass of the drug by appropriate analytical techniques. Because of that, the results are biased by the ununiform drug distribution within the MPs (e.g. the smallest MPs are excipient-only type).

**Figure 11.**
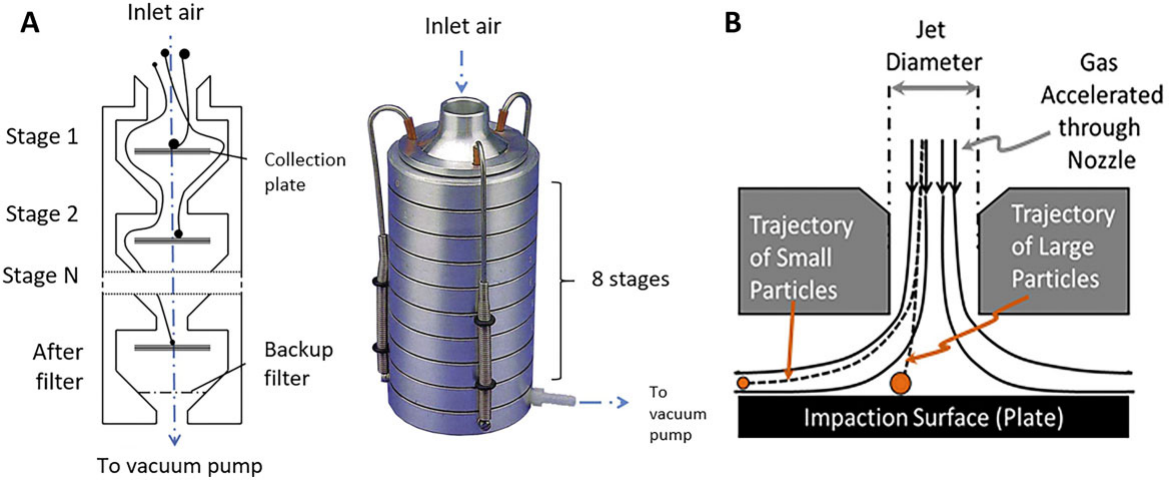
Scheme of a multistage cascade impactor (**A**) [151] and single stage (**B**) [152]. The pictures adapted with permission.

The most basic type of such equipment is a single-stage impactor. It consists of a nozzle plate containing one or more circular or slot-shaped jets (nozzles) of known diameter and distance from a collection surface (Fig. 11). The bigger particles tend to stick to the surface, whereas the smaller ones will change their trajectory due to the lower inertia of such MPs. In a multistage CI, MPs that do not stop at the first surface will proceed to a similar chamber with a thinner nozzle and a distance between the adsorber surface and the device walls. Typical CIs have around seven or more stages that are designed to separate MPs into aerodynamic sizes fractions [150]. Characteristically, the CI is used to quantify the respirable fraction or fine particle dose (usually the percentage of particles < 5 μm diameter) as an estimate of lung delivery [10].

CIs have been improving for many years now, but all devices are based on the same principle. The most common version of the CI present in current studies is the NGI [153]. Throughout the years, several studies have been performed comparing different CI approaches [154, 155]. They showed that although there are some differences in the results, all currently available devices may bring valuable results.

CI/NGI analysis may provide a sufficient prediction of the clinical performance of the formulation with high *in vitro* to *in vivo* correlation when the experimental setup is designed well to mimic the real situation parameters, like e.g. inflow rate. On the other hand, such tests are also limited by mimicking only the route of the formulation in the airways, not concerning its fate there. The final therapeutic performance is related to the other physicochemical and biological aspects as well (e.g. release kinetics, macrophage uptake, mucus penetration capability, etc.). [156]. This problem leads to the necessity of various biological tests.

Today, one of the biggest trends is to obtain highly porous MPs to increase their physical diameter so that alveolar macrophages cannot absorb them and to keep the aerodynamic diameter below 5 µm so that MPs can still reach the deep lungs. The most popular technique for this approach is to add the porosity generating agent to the water-1 phase by double emulsification. Currently, the most popular such agent seems to be ammonium bicarbonate. However, this method may be useful only for the manufacturing of polymeric MPs as a result of the ease with which this material can be formed into different shapes. Additionally, a large amount of MPs is always left in the preseparator, despite the chosen CI device. It may cause some problems in terms of repeatable dose delivery. Therefore, this field would also be encouraged to develop.

### *In vitro* tests

Typical *in vitro* tests on cell cultures generally only provide indicative information on biocompatibility, as they assess critical limits rather than a large amount of information on interactions between formulation and lung tissue [157]. The reason for this is that the lungs are a complex structure made up of more than 40 cell types, each cell type playing a specific role while interacting with the other ones. Therefore, investigating the formulation with only one type of cell culture may show to some extent whether the composition is cytotoxic but does not guarantee the cytocompatibility in, e.g. lower concentrations. One of the possible approaches to increase the reliability of *in vitro* results is to use advanced models based on cocultures of two or three cell types. In such circumstances, the ability to observe the interaction between different cells may provide additional information about whether the functions of cells are maintained correctly, not only if they are viable [158].

There are two types of cells to be used: primary cultures isolated from human or animal tissues or continuously growing, naturally or by genetic modification, cell lines. Although primary cells would mimic the clinical situation more efficiently, they are not commonly used due to their limited lifespan. Also, there are issues with availabilities, as obtaining them requires time-consuming isolation procedures. Therefore, the almost eternal lifespan of commercial cell lines is convenient. Such cells are either isolated from tumors—which have unlimited possibility of dividing naturally (e.g. A549)—or they are artificially immortalized primary cultures with the use of viral vectors (e.g. BEAS-2B). This type of cell is not only relatively easy to handle and widely available, but also allows one to compare the experiments provided anywhere in the world, as they are well standardized. On the other hand, both mutational and virial modifications lead to more or less significant changes in cell physiology. As a result, there is always a risk of inaccuracy in terms of mimicking natural conditions [159]. There are a few mechanisms of lung injury related to the specific cell type and their interactions. Furthermore, the mechanisms of the diseases are not easy to reproduce *in vitro* considering the complex structure of the lungs. A path to create a possible solution can be to model soft tissue in 3D. However, it also has a lot of limitations, because it is not yet possible to co-culture all the necessary cell types together. This is why the currently existing models focus on more simple injuries rather than complicated cases, e.g. lung fibrosis.

It is expected that the future models will apply the systems allowing for circulation of the media (using e.g. microfluidic devices). For this approach, the trend of organ-on-a-chip-based systems is becoming increasingly popular due to their potential to mimic the clinical situation to the greatest extent [160, 161]. This leads to the future possibility of connecting several chip units to mimic whole human body metabolism. It would allow investigation of, e.g. the influence of inhalable formulations metabolites on liver and kidneys, which is now only possible *in vivo* [162].

Another strategy to acquire more reliable results than typical cell culture tests that create some bridge between *in vitro* and *in vivo* experiments could be *ex vivo* tests on explanted living tissues coming from animals or human medical waste. This kind of study uses fresh living tissue where all types of cells are present and natural interactions between them are preserved. Such an analysis allows answering the question about not only if the formulation is cytotoxic but also evaluating the whole impact on the tissue in terms of, e.g. morphology, viability, gene expression, inflammation, etc. [163, 164], while the handling of *ex vivo* experiments is very similar to *in vitro* tests, once the tissue is possessed. This kind of analysis is currently used to investigate complicated pathological processes [165, 166] and may be successfully transferred to the investigation of the cytotoxicity of particle-based formulations, which is consistent with *in vitro* studies [167]. Naturally, the supply of explants is very limited because only fresh tissue can be used, so it can be obtained only by harvesting from sacrificed animal or human medical waste which acquisition, from obvious reasons, cannot be planned.

### Animal models in studies of drugs delivered directly to the lungs

**Figure 12.**
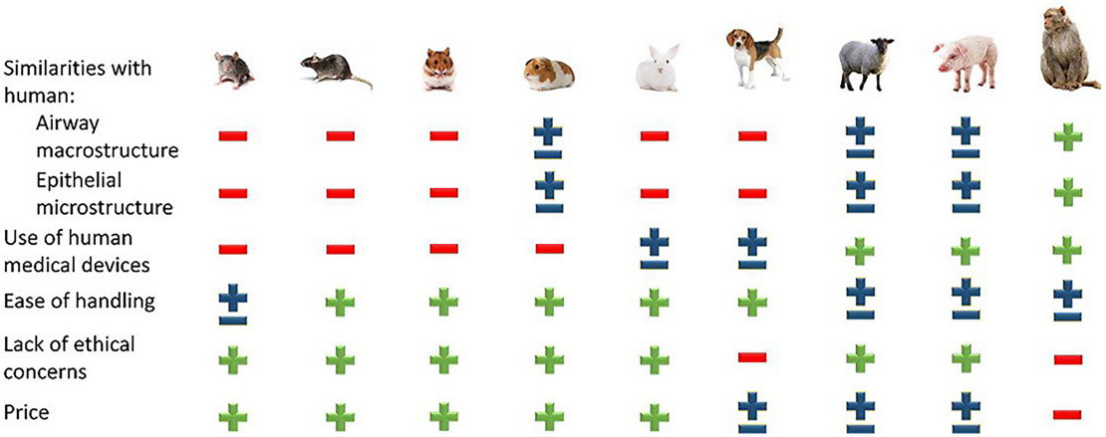
Advantages and disadvantages of using animal studies of pulmonary drug delivery to the lungs [169]. The picture adapted with permission.

Clinically relevant animal models are necessary for the investigation and development of therapeutics [168]. In a search for an effective treatment and validation of the therapies, different classes of animal models have been developed, which include both small (mouse, rat, hamster, guinea pig) and large animals (rabbit, dog, sheep, pig, monkey). These models have advanced our understanding of the mechanism of smoke inhalation injury, allowing a better understanding of the pathogenesis and pathophysiology as well as the development of new therapies. All animal models have limitations in replicating complex clinical conditions associated with smoke inhalation injury in humans (Fig. 12). Therefore, for a correct interpretation of the results and to avoid bias, a precise understanding of the similarities and differences of the lungs between different species of animals and humans is critical [169].

Almost all *in vivo* studies cited in this review were performed on small animals (e.g. rats) and the MPs were administrated intratracheally. Such an approach may give some promising results. However, it is far from being in compliance with the real clinical conditions. First, there is no rightful inhalation, so many patient-related parameters are not considered. Second, the anatomy of their airways is very different from that of humans. Both of these factors can lead to misinterpretation and result in poorer clinical performance of the formulation, as assumed. The main problem is the lack of a sufficient animal model for inhalation studies. Even ignoring the increased cost and handling issues of the bigger animals, of which the airway microstructure and macrostructure are similar to humans, there is still a problem of not being able to teach an animal to use the DPI device as a human would. It is a serious problem, as we lack a representative stage between *in vivo* studies and clinical ones, which is not an issue, e.g. testing scaffolds for tissue engineering and regeneration.

### Clinical trials

According to ClinicalTrials.gov [170], there are currently only 294 clinical trials of different therapies using DPIs at any stage worldwide. Compared to the number of published scientific papers in the field—Google Scholar suggests [171] 14 500 papers related to the abbreviation DPI in 2022 only—it shows how few of the investigated solutions can make it to the clinic. It is the consequence of the number of challenges faced in designing an effective DPI formulation.

Although it is not easy to reach the clinical stage of the DPI investigation, approaching this stage does not guarantee any success. It happens very often that the results do not meet the prespecified end points [172], so, in fact, only some of the solution studied in clinics will be transferred to everyday use. Furthermore, the results of the clinical study may be biased by inadequate use, since only 11.9% of studies provide reliable information on the training of patients to properly use inhaler devices properly [173]. To enhance the chances of different investigated formulations becoming real therapy methods and not only for the purpose of research for curiosity, sufficient communication between clinics and research institutes is required. The art of clinical trials is complicated and based on statistical analysis to possibly limit the influence of individual variability of patients and will provide trustworthy results only with the right and thoughtful setup. The correct use of DPI or other inhalers is strongly related to the outcoming therapeutic efficacy. Therefore, ensuring the right exploitation by patients should never be overlooked, not to negatively bias the results in the very last step to the everyday use.

## Conclusion

In this review article, we present an overview of novel dry powder MPs based on different materials such as polysaccharides, polymers and lipids. All of these types of dry powders have the potential to be approved for clinical use. However, it is a laborious and long-lasting process to achieve that. Creating a new DPI formulation requires the involvement of many different specialists, from engineers to clinicians. There are several stages in manufacturing such a formulation, and each of them has its own requirements that are often difficult to meet simultaneously.

The problems faced predominantly relate to the differences between the physical and aerodynamic size of the particles and the fact that the right size for inhalation is at the same time the easiest for macrophages to phagocytose. Furthermore, the complexity of the environment of the human lung makes it extremely difficult to mimic the real conditions in the lab. On the other hand, the lack of a precise animal model for *in vivo* studies leads to limited success of clinical trials that already are a distinction to only the most promising formulations.

To overcome difficulties, several approaches are used. First, most of the polymeric MPs are obtained by modified emulsification, which allows them to be made porous. Due to this, the aerodynamic diameter remains within the range of 1–5 µm range; however, the physical diameter increases to up to 20 µm sufficiently avoiding macrophage uptake. Additionally, composite MPs consisting of NPs that are quickly redispersed after reaching the lungs allow the mucus to penetrate sufficiently after reaching the side of action. It is especially interesting because both used materials can be adjusted to a specific role: high drug loading capacity and proper release kinetics for NPs and the right aerodynamic properties for MPs, respectively. In addition, there are several techniques to fill the gap between classic *in vitro* and *in vivo* studies, such as 3D cell cultures or *ex vivo* tissue analysis, to make the approval process more consistent and controlled. These approaches have been appearing mainly in recent publications, indicating promising trends for future DPIs.

In light of this, it is and will continue to be a long and demanding journey to implement a new DPI formulation in everyday therapies. However, novel approaches that use the development of recent knowledge on lung diseases and modern technological solutions will provide many beneficial DPI formulations for medical use.
